# Access to Enantiomerically Pure *P*-Chiral 1-Phosphanorbornane Silyl Ethers

**DOI:** 10.3390/molecules28176210

**Published:** 2023-08-23

**Authors:** Kyzgaldak Ramazanova, Soumyadeep Chakrabortty, Fabian Kallmeier, Nadja Kretzschmar, Sergey Tin, Peter Lönnecke, Johannes G. de Vries, Evamarie Hey-Hawkins

**Affiliations:** 1 Institute of Inorganic Chemistry, Faculty of Chemistry and Mineralogy, Leipzig University, Johannisallee 29, 04103 Leipzig, Germany; kyzgaldak.ramazanova@yahoo.com (K.R.); nadja-kretzschmar@gmx.de (N.K.); loenneck@rz.uni-leipzig.de (P.L.); 2 Leibniz Institute for Catalysis (LIKAT), Albert-Einstein-Straße 29A, 18059 Rostock, Germany; soumyadeep.chakrabortty@catalysis.de (S.C.); fabian.kallmeier@anu.edu.au (F.K.); sergey.tin@catalysis.de (S.T.); johannes.devries@catalysis.de (J.G.d.V.)

**Keywords:** asymmetric hydrogenation, enantiopure, *P*-chiral phosphines, silylation

## Abstract

Sulfur-protected enantiopure *P*-chiral 1-phosphanorbornane silyl ethers **5a**,**b** are obtained in high yields via the reaction of the hydroxy group of *P*-chiral 1-phosphanorbornane alcohol **4** with *tert*-butyldimethylsilyl chloride (TBDMSCl) and triphenylsilyl chloride (TPSCl). The corresponding optically pure silyl ethers **5a**,**b** are purified via crystallization and fully structurally characterized. Desulfurization with excess Raney nickel gives access to bulky monodentate enantiopure phosphorus(III) 1-phosphanorbornane silyl ethers **6a**,**b** which are subsequently applied as ligands in iridium-catalyzed asymmetric hydrogenation of a prochiral ketone and enamide. Better activity and selectivity were observed in the latter case.

## 1. Introduction

**Figure 1:**
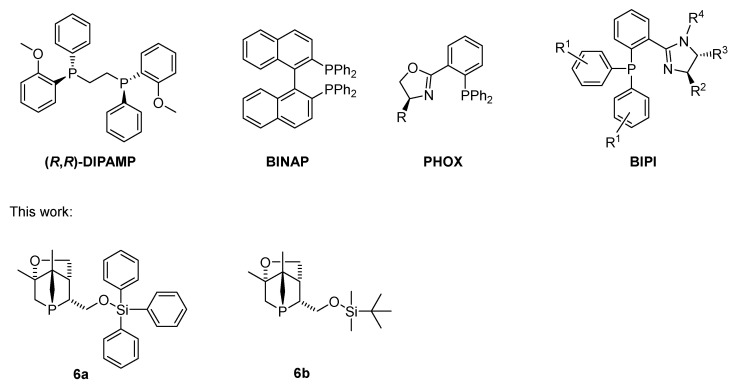
Previously reported chiral phosphines (**top row**) and the *P*-chiral 1-phosphanorbornane silyl ethers **6a**,**b** (**bottom row**) reported in this work.

Chiral phosphines play a pivotal role in asymmetric homogeneous catalysis [1,2,3,4,5,6,7,8]. *P*-stereogenic phosphines, a special class of chiral phosphines, have been well established in catalysis ever since the pioneering work on asymmetric hydrogenation (AH) employing a *P*-chiral ligand was introduced by Horner et al. [9] and Knowles et al. [10]. The development of the privileged *P*-chiral ligand (ethane-1,2-diyl)bis[(2-methoxyphenyl)(phenyl)phosphane] (DIPAMP) by Knowles and coworkers [11] led to the first industrial asymmetric hydrogenation in the production of the drug l-3,4-dihydroxyphenylalanine (l-DOPA) used in the treatment of Parkinson’s disease [12] (Figure 1). A few years later, Noyori, another Nobel prize winner, and his group developed the axially chiral phosphine 2,2′-bis(diphenylphosphino)-1,1′-binaphthyl (BINAP) [13] and showed that complexes with ruthenium were effective in asymmetric hydrogenations of a wide range of olefins and carbonyl compounds [14,15,16,17]. Nowadays, AH is considered one of the most important enantioselective syntheses that gives access to many important optically active compounds. Among the widely used metals in such reactions, the iridium-catalyzed hydrogenations have been extensively studied [18,19,20,21,22]. The symmetric Crabtree catalyst [23], the chiral P,N bidentate PHOX ligand developed by Pfaltz et al. [24,25,26] and BIPI ligands by Busacca et al. [27] are key examples of Ir-based catalysts in hydrogenation reactions. The majority of the developed procedures employ bidentate hetero-donor P,X (X = N, O) ligands as they were believed to considerably influence enantioselectivities due to better chirality transfer [28,29,30,31]. Thus, the reluctance to employ monodentate ligands in AH is understandable, especially given the proven history of success with chelate ligands. However, evidence of high enantiomeric excess (*ee*) values in AH achieved using monodentate ligands has been reported [32,33,34,35]. Despite the success of the published compounds in enantioselective catalysis, industry and academia are still searching for better, more efficient and sustainable catalysts, resulting in a number of new *P*-chiral compounds being reported regularly [36,37,38].

**Scheme 1:**
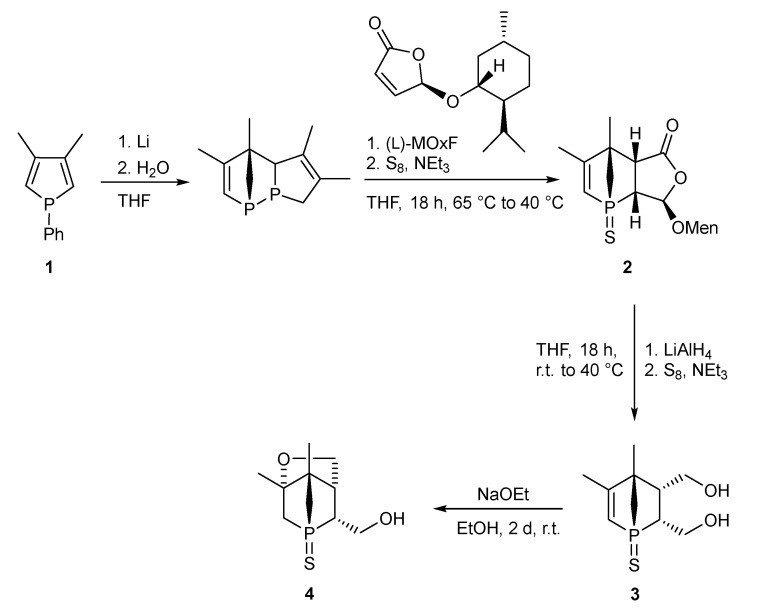
Preparation of 1-phosphanorbornane alcohol **4**.

Phospholes are known to undergo hetero-Diels–Alder (HDA) reactions with various dienophiles to afford P-heterocyclic compounds [39], and recently, we reported the unprecedented phospha-aza-Diels–Alder reaction using an N-sulfonyl α-imino ester to produce 1-phospha-2-azanorbornenes (PANs) [40]. We also showed that the reactive P–N bond of PANs can be cleaved by both achiral and enantiopure nucleophiles to yield racemic 2,3-dihydrophosphole and optically pure 1-alkoxy-2,3-dihydrophosphole derivatives, respectively [40,41]. Moreover, the reduction of PAN with lithium aluminum hydride (LAH) resulted in a seven-membered P-heterocycle [42]. Previously, we reported the first stereoselective HDA reaction between (5*R*)-(l-menthyloxy)-2(5*H*)-furanone (MOxF) and 2*H*-phospholes (Scheme 1) to produce *P*-chiral 1-phosphanorbornenes (**2**) [43] as well as *P*-chiral 7-phosphanorbornenes [44] in high yields. Moreover, the reduction of 1-phosphanorbornenes yields access to 1-phosphanorbornene diol **3**. The latter undergoes an intramolecular Michael addition to afford 1-phosphanorbornane alcohol **4**, which can be converted into enantiopure 1-phosphanorbornane bromide for subsequent functionalization [45].

Herein, we report the one-step synthesis of enantiomerically pure *P*-stereogenic 1-phosphanorbornane silyl ethers obtained via reaction of the hydroxy group in **4** with chlorosilanes followed by desulfurization. The application of these ligands in iridium-catalyzed AH of prochiral enamides, namely methyl-(*Z*)-α-acetamidocinnamate (MAC), was studied. To our knowledge, such ligands have not yet been tested in AH nor any other enantioselective homogeneous catalysis.

## 2. Results and Discussion

### 2.1. Synthesis and Characterization of ***5a**,**b***

**Scheme 2:**
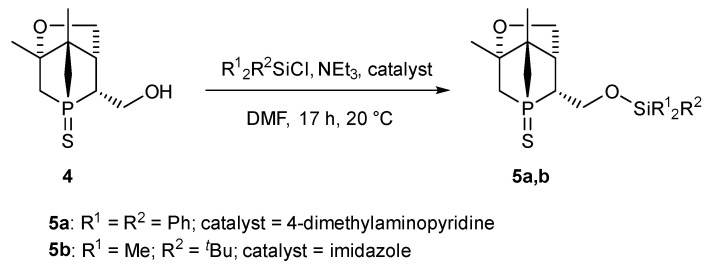
Synthesis of sulfur-protected 1-phosphanorbornane silyl ethers **5a**,**b**.

The enantiopure 1-phosphanorbornane alcohol **4** (PNA) is readily prepared in very good yields [45]. The *P*-chiral 1-phosphanorbornane silyl ethers **5a**,**b** are obtained by reaction of PNA **4** with chlorosilanes in dimethylformamide (DMF) in the presence of base and catalyst (Scheme 2). The formation of silyl ethers is widely exploited for the protection of alcohols, and numerous suitable silylation reagents have been reported [46,47,48,49]. We selected *tert*-butyldimethylsilyl chloride (TBDMSCl) and triphenylsilyl chloride (TPSCl), as the corresponding bulky siloxy groups provide high stability in acidic and basic media compared to the less sterically demanding trimethylsilyl or triethylsilyl ethers [49,50].

In this kind of established reaction, the choice of catalyst, solvent and base is important. Initially, when CH_2_Cl_2_ was used as the solvent, the reaction of **4** with TPSCl was much slower compared to DMF as the solvent. This supports the reported evidence of DMF acting as a catalyst itself in silylation reactions of alcohols [51]. Consistent with the classical procedure developed by Corey et al. [52], imidazole was employed as catalyst to afford **5b**, while for the reaction with TPSCl, 4-dimethylaminopyridine (DMAP) was used as it was previously reported to be a successful catalyst.

Stirring at 20 °C overnight resulted in full consumption of PNA as confirmed by ^31^P{^1^H} NMR spectroscopy (CDCl_3_, singlet at 43.4 ppm for **5a** and 43.6 ppm for **5b**). Thus, this one-step procedure gives access to **5a**,**b** in very good yields under mild conditions. Pure **5a**,**b** were isolated by crystallization; single crystals suitable for X-ray crystallo-graphy (Supplementary Materials, Section S3) were obtained by dissolving **5a**,**b** in a hot *^i^*PrOH/*n*-hexane mixture and cooling to −25 °C for 17 h. High chemical (98%) and optical purity of the UV-active compound **5a** were confirmed by HPLC using a chiral column (Supplementary Materials, Figure S13), while the chemical purity of **5b** was verified by elemental analysis. High-resolution mass spectrometry (HRMS) showed the presence of the expected ions, namely [**5a** + H]^+^ (*m*/*z* 491.1617), [**5a** + NH_4_]^+^ (*m*/*z* 508.1878), and [**5a** + Na]^+^ (*m*/*z* 513.1447) or [**5b** + H]^+^ (*m*/*z* 347.1631) and [**5b** + Na]^+^ (*m*/*z* 369.1451), respectively. The molecular structures of **5a**,**b** were also confirmed by 2D NMR spectroscopy.

**Figure 2:**
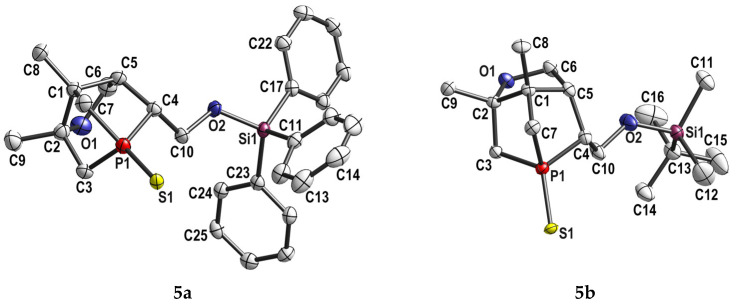
Molecular structures of **5a** and **5b**. Hydrogen atoms were omitted for clarity. Only one of the two independent molecules of **5a** is shown. Displacement ellipsoids are drawn at the 50% probability level.

The enantiopure compounds crystallize in the triclinic space group *P*1 with two independent molecules in the unit cell (**5a**) or in the monoclinic space group *P*2_1_ with Z = 2 (**5b**), respectively. The phosphorus atom has a distorted tetrahedral environment (Figure 2). The Si–O bond lengths are in the range of 164.1(2) to 165.4(2) pm, which is in agreement with the literature [53,54].

### 2.2. Desulfurization of Compounds ***5a**,**b***

**Scheme 3:**
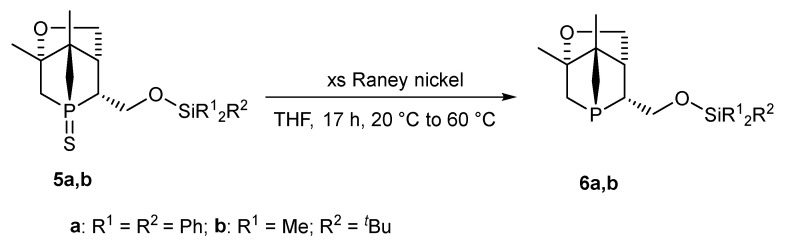
Desulfurization of **5a**,**b** with excess Raney nickel.

The *P*-chiral 1-phosphanorbornane silyl ethers **5a**,**b** can be reduced (desulfurized) to the corresponding phosphorus(III) derivatives with excess of freshly activated Raney nickel at room temperature (Scheme 3). No further work up is required after the reaction is finished. Moreover, this method is mild and tolerates many other functional groups guaranteeing selective desulfurization of the phosphorus atom. In contrast, treating **5a**,**b** with the very strong base lithium aluminum hydride (LAH) at 50 °C requires further quenching and has a risk of side reactions. Nevertheless, ^31^P{^1^H} NMR spectra (CDCl_3_) of the reaction mixtures of **5a** and both reducing agents revealed full conversion of the starting material and formation of **6a** (singlet at −45.9 ppm). In contrast, **5b** can only be reduced cleanly with excess Raney nickel (singlet at −46.3 ppm for **6b** in the ^31^P{^1^H} NMR spectrum (CDCl_3_)), while the reduction of **5b** with LAH resulted in formation of side products, which are presumably formed by deprotection of the silyl group. Although the TBDMS and TPS groups are known to be stable in various media, examples of TBDMS ether cleavage by LAH have been reported previously [55,56,57]. Therefore, the reduction of both compounds was carried out with Raney nickel.

The structures of **6a**,**b** were fully confirmed by 2D NMR spectroscopy. However, due to the high oxophilicity of the phosphorus atom, mainly the corresponding oxides were observed by HRMS ([**6a** + O + H]^+^ (*m*/*z* 475.1795), [**6a** + O + Na]^+^ (*m*/*z* 497.1644), [**6b** + Na]^+^ (*m*/*z* 337.1722), [**6b** + O + Na]^+^ (*m*/*z* 353.1668), and [**6b** + O + K]^+^ (*m*/*z* 369.1420)).

## 3. Catalysis

**Scheme 4:**
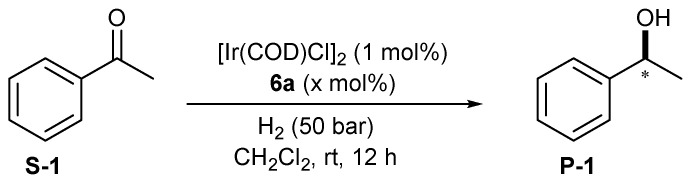
Ir-catalyzed asymmetric hydrogenation of acetophenone using **6a** as ligand (* indicates a chiral center).

Bidentate (mixed donor) chiral ligands developed by Pfaltz et al. [58] and Andersson et al. [59] are mostly used in Ir-catalyzed asymmetric hydrogenation of olefins. On the other hand, the use of chiral monodentate phosphines in Ir-catalyzed enantioselective hydrogenation is uncommon. Encouraged by the previous result on an Ir/phosphoramidite catalyst in AH [34], we evaluated the activity of the bulky monodentate *P*-chiral 1-phosphanorbornane silyl ethers **6a**,**b** in the asymmetric hydrogenation of carbonyl compounds and olefins. No or minor conversion was observed in the asymmetric hydrogenation of acetophenone (**S-1**) using [Ir(COD)Cl]_2_/**6a** (1 mol%, M:L = 1:3) as catalyst in dichloromethane (Scheme 4). However, up to 20% conversion was obtained with potassium *tert*-butoxide as base (20 mol%), albeit with formation of racemic 1-phenylethan-1-ol (**P-1**) (Table 1).

**Scheme 5:**
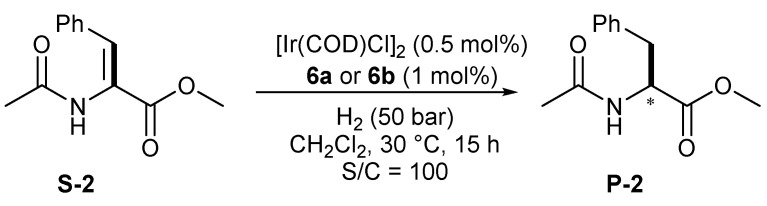
Asymmetric hydrogenation of methyl (*Z*)-2-acetamido-3-phenylacrylate (* indicates a chiral center).

Then, the catalytic activities of **6a**,**b** in the asymmetric hydrogenation of the functionalized olefin methyl (*Z*)-2-acetamido-3-phenylacrylate as benchmark substrate was studied. The catalytic experiments were performed by premixing the ligand (**6a** or **6b**) and the iridium complex (Scheme 5). The hydrogenation of **S-2** proceeds with 98% conversion using [Ir(COD)Cl]_2_/**6a** (5 mol%, M:L = 1:1) as the catalyst, but with poor enantioselectivity (Table 2, Entry 1). A similar activity was observed when the catalyst loading was decreased to 0.5 mol% (Table 2, Entry 2) in dichloromethane. Changing the solvent to MeOH and THF did not improve the *ee*, but resulted in lower conversion (Table 2, Entry 3 and 4). The catalytic activity was not affected by altering the silyl substituent from SiPh_3_ (**6a**) to SiMe_2_*^t^*Bu (**6b**) (Table 2, Entry 5). Apparently, the bulky silyl group is not in close proximity to the catalytically active iridium center.

## 4. Conclusions

A highly efficient and facile synthesis of enantiomerically pure sulfur-protected *P*-stereogenic 1-phosphanorbornane silyl ethers **5a**,**b** via reaction of the alcohol function of **4** with chlorosilanes is described. Moreover, this method can be applied to prepare a variety of compounds with desired electronic and steric effects via the appropriate choice of the corresponding chlorosilane. The phosphorus(III) derivatives **6a**,**b** are readily accessible via desulfurization of **5a**,**b** with excess Raney nickel. The phosphines **6a**,**b** were tested as ligands in the Ir-catalyzed asymmetric hydrogenation of acetophenone and methyl (*Z*)-2-acetamido-3-phenylacrylate resulting in moderate to high conversions but poor *ee*. Further studies on different ligand variations based on the chiral phosphanorbornane motif and their application in enantioselective catalysis are underway.

## 5. Materials

### 5.1. General Information

All air-sensitive reactions were carried out under dry high purity nitrogen using standard Schlenk techniques. THF was degassed and distilled from potassium. DMF was degassed and dried under activated 4 Å molecular sieves. TBDMSCl and TPSCl were purchased from Carbolution (St. Ingbert, Germany) or Sigma Aldrich (St. Louis, MO, USA), respectively. The NMR spectra were recorded with a Bruker Avance DRX 400 spectrometer (^1^H NMR 400.13 MHz, ^13^C NMR 100.63 MHz, ^31^P NMR 161.98 MHz) or a Bruker Fourier 300 spectrometer (^1^H NMR 300.23 MHz, ^13^C NMR 75.50 MHz). ^13^C{^1^H} NMR spectra were recorded as APT spectra. The assignment of the chemical shifts and configurations was performed using correlation spectroscopy (COSY) and heteronuclear single quantum coherence (HSQC) techniques. Tetramethylsilane (TMS) was used as the internal standard in the ^1^H NMR spectra and all other nuclei spectra were referenced to TMS using the Ξ-scale [60]. The numbering scheme of **5a**,**b** and **6a**,**b** is given in the Supplementary Materials. High-resolution mass spectra (HRMS; electrospray ionization (ESI)) were measured using a Bruker Daltonics APEX II FT-ICR spectrometer (Billerica, MA, USA). IR spectra were obtained with an FTIR spectrometer (Nicolet iS5 FTIR by Thermo Scientific, Waltham, MA, USA) in the range of 400–4000 cm^−1^ in KBr. Column chromatography was performed using silica 60 (0.015–0.040 mm) purchased from Merck (Rahway, NJ, USA). UV light (389 nm) and iodine (saturated atmosphere) were used as staining reagents. The synthesis of the starting material PNA **4** and Raney nickel activation were carried out according to the literature [45].

### 5.2. Synthesis

#### 5.2.1. Synthesis of **5a**

TPSCl (0.38 g, 1.28 mmol) was added to a solution of **4** (0.2 g, 0.86 mmol) and NEt_3_ (0.18 mL, 1.28 mmol) in 12 mL DMF at room temperature. Further 20 mg of DMAP (0.017 mmol) was added and the reaction mixture was stirred for 17 h at 20 °C. The mixture was washed with sat. aq. NH_4_Cl solution and the separated organic layer was further washed with 5 mL water 3 times. The combined organic phases were dried over MgSO_4_. The solvent was removed under reduced pressure to give a white powder. The compound was dissolved in hot *^i^*PrOH/*n*-hexane and then cooled at −25 °C for 17 h. The formed white solid was isolated, washed with 3 mL cold *n*-hexane 3 times and dried in vacuo to afford 308 mg of **5a** as a white powder. Yield: 308 mg (73%). Single crystals of **5a** suitable for X-ray crystallographic studies were obtained by dissolving **5a** in a hot *^i^*PrOH/*n*-hexane mixture and cooling to −25 °C for 17 h (Supplementary Materials, Figure S14).

^1^H NMR (400 MHz, CDCl_3_): δ 7.61 (m, 5H), 7.50–7.25 (m, 10H), 4.33 (m, 1H, H-5 or H-6a), 4.16–4.01 (m, 2H), 3.94 (dd, *J* = 9.9, 5.7 Hz, 1H, H-5 or H-6a), 2.67–2.45 (m, 2H), 2.25 (m, 1H, H-6 or H-7 or H-2), 2.00 (m, 1H), 1.92–1.78 (m, 2H), 1.24 (s, 3H, H-3a or H-4a), 1.20 (s, 3H, H-3a or H-4a) ppm; ^13^C{^1^H} NMR (101 MHz, CDCl_3_): δ 135.4 (s, C-aryl), 135.2 (s, C-aryl), 133.4 (s, C-aryl quart.), 130.3 (s, C-aryl), 129.8 (s, C-aryl), 128.0 (s, C-aryl), 127.7 (s, C-aryl), 86.3 (s, C-quart.), 66.3 (s), 59.0 (d, *J*_C,P_ = 6.1 Hz), 51.3 (d, ^2^*J*_C,P_ = 19.5 Hz, C-quart.), 47.3 (d, ^2^*J*_C,P_ = 2 Hz, C-5), 44.8 (d, ^1^*J*_C,P_ = 46.7 Hz, C-6), 41.4 (d, ^1^*J*_C,P_ = 44.8 Hz, C-2 or C-7), 40.3 (d, ^1^*J*_C,P_ = 51.8 Hz, C-2 or C-7), 23.9 (d, ^3^*J*_C,P_ = 7.2 Hz, C-3a or C-4a), 18.3 (d, ^3^*J*_C,P_ = 15.9 Hz, C-3a or C-4a) ppm; ^31^P{^1^H} NMR (162 MHz, CDCl_3_): δ 43.4 (s) ppm; HRMS (ESI, MeCN), *m*/*z*: found: 491.1617, calculated for [M + H]^+^: 491.1624; found: 508.1878, calc. for [M + NH_4_]^+^: 508.1890; found: 513.1447, calc. for [M + Na]^+^: 513.1444; found: 998.3474, calc. for [2M + NH_4_]^+^: 998.3441; found: 1003.3032, calc. for [2M + Na]^+^: 1003.2996; IR (KBr, $\tilde{v}$/cm^−1^): 3067 (w), 2975 (w), 2881 (w), 1588 (w), 1485 (w), 1427 (m), 1381 (w), 1369 (w), 1306 (w), 1250 (w), 1189 (w), 1114 (s), 1077 (s), 1053 (m), 1042 (m), 1012 (m), 996 (m), 958 (m), 928 (w), 881 (m), 862 (w), 841 (w), 800 (m), 775 (m), 738 (m), 709 (s), 697 (s), 675 (m), 619 (m), 609 (w), 582 (w), 506 (s), 481 (s), 448 (m), 435 (m).

#### 5.2.2. Synthesis of **5b**

TBDMSCl (0.146 g, 0.97 mmol) was added to a solution of **4** (0.15 g, 0.65 mmol) and NEt_3_ (0.135 mL, 0.97 mmol) in 10 mL DMF at room temperature. Further 13 mg of imidazole (0.19 mmol) were added and the reaction mixture was stirred for 17 h at 20 °C. The mixture was washed with sat. aq. NH_4_Cl solution and the separated organic layer was further washed with 5 mL water 3 times. The combined organic phases were dried over MgSO_4_. The solvent was removed under reduced pressure to give a white powder. The compound was dissolved in hot *^i^*PrOH/*n*-hexane and then cooled at −25 °C for 17 h. The resulting white solid was washed with 3 mL cold *n*-hexane 3 times and dried in vacuo to afford 154 mg of **5b** as a white powder. Yield: 154 mg (69%). Elemental analysis: C_16_H_31_O_2_PSSi (346.54) calc. C 55.5%, H 9.0%; found C 55.7%, H 9.1%. Single crystals of **5b** suitable for X-ray crystallographic studies were obtained by dissolving **5b** in a hot *^i^*PrOH/*n*-hexane mixture and cooling to −25 °C for 17 h (Supplementary Materials, Figure S15).

^1^H NMR (400 MHz, CDCl_3_): δ 4.20–4.07 (m, 2H, H-5a/6a), 3.94 (m, 2H, H-5a/6a), 2.62–2.41 (m, 2H, H-5 or H-6), 2.31 (m, 1H, H-5 or H-6), 2.02 (m, 1H, H-2 or H-7), 1.96–1.83 (m, 2H, H-7 or H-2), 1.26 (s, 3H, H-3a or H-4a), 1.20 (s, 3H, H-3a or H-4a), 0.88 (s, 9H, H-9a), 0.09 (s, 6H, H-8) ppm; ^13^C{^1^H} NMR (101 MHz, CDCl_3_): δ 86.3 (s, C-quart.), 66.2 (s, C-5a), 58.1 (d, ^2^*J*_C,P_ = 5.6 Hz, C-6a), 51.3 (d, ^2^*J*_C,P_ = 19.4 Hz, C-quart.), 47.3 (d, ^2^*J*_C,P_ = 2.3 Hz, C-5), 44.8 (d, ^1^*J*_C,P_ = 47.0 Hz, C-6), 41.6 (d, ^1^*J*_C,P_ = 44.8 Hz, C-2 or C-7), 40.3 (d, ^1^*J*_C,P_ = 51.8 Hz, C-2 or C-7), 25.8 (s, C-9a), 23.9 (d, ^3^*J*_C,P_ = 7.3 Hz, C-3a or C-4a), 18.3 (d, ^3^*J*_C,P_ = 16.0 Hz, C-3a or C-4a), 18.1 (s, C-9), −5.5 (d, *J* = 6.1 Hz, C-8) ppm; ^31^P{^1^H} NMR (162 MHz, CDCl_3_) δ 43.6 (s) ppm; HRMS (ESI, MeCN), *m*/*z*: found: 347.1631, calc. for [M + H]^+^: 347.1624; found: 369.1451, calc. for [M + Na]^+^: 369.1444; IR (KBr, $\tilde{v}$/cm^−1^): 2948 (m), 2925 (m), 2877 (m), 2853 (m), 1497 (w), 1468 (w), 1426 (w), 1383 (w), 1360 (w), 1311 (w), 1258 (m), 1245 (m), 1198 (w), 1162 (w), 1122 (m), 1077 (s), 1041 (m), 1029 (m), 1011 (m), 959 (m), 930 (w), 881 (s), 867 (s), 829 (m), 815 (m), 783 (s), 768 (s), 749 (m), 721 (s), 675 (s), 658 (s), 586 (w), 563 (w), 511 (m), 481 (w), 447 (m).

#### 5.2.3. Synthesis of **6a**

Compound **5a** (200 mg, 0.41 mmol) was added to a suspension of freshly activated Raney nickel in THF (ca. 2 g, excess) and stirred for 17 h at room temperature. The clear solution was filtered and the black solid was washed four times with 5 mL THF each. The solution was concentrated to give 142 mg of **6a** as a white solid (76%). Yield: 142 mg (76%).

^1^H NMR (400 MHz, THF-d_8_): δ 7.54–7.44 (m, 3H), 7.39–7.33 (m, 3H), 7.33–7.19 (m, 6H), 7.13 (m, 3H), 3.86 (m, 2H, H-2 or H-7), 3.61–3.51 (m, 2H, H-5a or H-6a), 2.38 (m, 1H, H-6 or H-5), 2.13 (m, 1H, H-5 or H-6), 1.44–1.16 (m, 4H, H-2 or H-7, H-5a or H-6a), 0.99 (s, 3H, H-3a or H-4a), 0.97 (s, 3H, H-3a or H-4a) ppm; ^13^C{^1^H} NMR (101 MHz, THF-d_8_): δ 135.1 (s, C-aryl), 134.9 (s, C-aryl), 134.2 (s, C-aryl quart.), 129.6 (s, C-aryl), 129.5 (s, C-aryl), 127.5 (s, C-aryl), 127.4 (s, C-aryl), 86.9 (s, C-quart.), 63.7 (s), 62 (d, ^1^*J*_C,P_ = 14.9 Hz, C-2 or C-7), 47.5 (d, ^2^*J*_C,P_ = 3.3 Hz, C-5), 45.1 (d, ^1^*J*_C,P_ = 13.5 Hz, C-6), 38 (d, ^1^*J*_C,P_ = 16.0 Hz, C-2 or C-7), 36.7 (d, *J* = 6.6 Hz, C-6a or C-5a), 23.7 (s, C-3a or C-4a), 17.5 (s, C-3a or C-4a) ppm; ^31^P{^1^H} NMR (162 MHz, C_6_D_6_): δ −45.6 (s) ppm; HRMS (ESI, MeCN), *m*/*z*: found: 475.1795, calc. for [M + O + H]^+^: 475.1863; found: 497.1644, calc for [M + O + Na]^+^: 497.1672.

#### 5.2.4. Synthesis of **6b**

Compound **5b** (53 mg, 0.153 mmol) was added to a suspension of freshly activated Raney nickel in THF (ca. 0.44 g, excess) and stirred for 17 h at room temperature. The clear solution was filtered and the black solid was washed four times with 2 mL THF each. The solution was concentrated to give 31 mg of **6b** as a colorless oil (63%). Yield: 31 mg (63%).

^1^H NMR (400 MHz, C_6_D_6_): δ 3.89–3.82 (m, 2H), 3.77–3.67 (m, 2H), 2.34 (m, 1H), 1.95–1.90 (m, 1H), 1.75 (dt, *J* = 15.4, 3.1 Hz, 1H, H-2 or H-7), 1.43–1.33 (m, 1H), 1.21–1.15 (m, 1H), 1.1 (s, 3H, H-3a or H-4a), 0.91 (s, 9H, H-8a), 0.89 (s, 3H, H-3a or H-4a), 0.29 (s, 6H, H-7) ppm; ^13^C{^1^H} NMR (101 MHz, C_6_D_6_): δ 64.1(s, C-5a), 61.3 (d, *J* = 15.0 Hz, C-6a), 47.6 (d, ^2^*J*_C,P_ = 3.6 Hz, C-5), 45.2 (d, ^1^*J*_C,P_ = 12.9 Hz, C-6), 38.52 (d, ^1^*J*_C,P_ = 15.7 Hz, C-2 or C-7), 37.03 (d, ^1^*J*_C,P_ = 6.4 Hz, C-2 or C-7), 25.7 (s, C-9a), 24.5 (s, C-3a or C-4a), 18.1 (s, C-3a or C-4a), −5.7 (d, *J* = 11.6 Hz, C-8) ppm; ^31^P NMR (162 MHz, C_6_D_6_): δ −45.6 (s) ppm; HRMS (ESI, MeCN), *m*/*z*: found: 337.1722, calc. for [M + Na]^+^: 337.1723; found: 353.1668, calc. for [M + O + Na]^+^: 353.1672; found: 369.1420, calc. for [M + O + K]^+^: 369.1412.

### 5.3. Catalysis

#### General Procedure for Hydrogenations

Ketone hydrogenation: The hydrogenation experiments were performed in stainless steel autoclaves charged with an insert suitable for up to 8 reaction vessels (4 mL) with teflon mini stirring bars. In a typical experiment, a reaction vessel was charged with [Ir(COD)Cl]_2_ (1 mol%), ligand (1–3 mol%, as desired) and base (20 mol%) and stirred for 10–15 min in the dichloromethane (2 mL). Then, acetophenone (**S-1**, 0.5 mmol) was added to the reaction vials maintaining the inert atmosphere and the vessels were placed in a high pressure autoclave. The autoclave was purged two times with nitrogen and three times with hydrogen. Finally, it was pressurized at 50 bar H_2_ at 25 °C for 12 h. Afterwards, the autoclave was depressurized and the contents of the reaction vessels were diluted with EtOAc and filtered through a short pad of silica. The conversion was determined by GC, GC-MS and NMR measurement and the enantiomeric excess was measured by chiral GC analysis.

Olefin hydrogenation: The hydrogenation experiments were performed in stainless steel autoclaves charged with an insert suitable for up to 8 reaction vessels (4 mL) with teflon mini stirring bars. In a typical experiment, a reaction vessel was charged with [Ir(COD)Cl]_2_ (0.5 mol%), ligand (1 mol%) in the appropriate solvent (2 mL). Then, methyl (*Z*)-2-acetamido-3-phenylacrylate (**S-2**, 0.5 mmol) was added to the reaction vials maintaining the inert atmosphere and the vessels were placed in a high pressure autoclave. The autoclave was purged two times with nitrogen and three times with hydrogen gas. Finally, it was pressurized at 50 bar H_2_ at 30 °C for 15 h. Afterwards, the autoclave was depressurized and the contents of the reaction vessels were diluted with EtOAc and filtered through a short pad of silica. The conversion was determined by GC, GC-MS and NMR measurements and the enantiomeric excess was measured by chiral GC analysis.

### 5.4. X-ray Crystallography Data

The data were collected on a Gemini diffractometer (Rigaku Oxford Diffraction) using Mo-Kα radiation and ω-scan rotation. Data reduction was performed with CrysAlisPro [61] including the program SCALE3 ABSPACK for empirical absorption correction. All structures were solved by dual space methods with SHELXT [62] and the refinement was performed with SHELXL [63]. For **5b**, hydrogen atoms were calculated on idealized positions using the riding model, whereas for **5a**, a difference-density Fourier map was used to locate hydrogen atoms. Structure figures were generated with DIAMOND-4 [64].

CCDC deposition numbers 2287331 for **5a** and 2287332 for **5b** contain the supplementary crystallographic data for this paper. These data can be obtained free of charge via https://www.ccdc.cam.ac.uk/structures/, accessed on 25 May 2023 (or from the Cambridge Crystallographic Data Centre, 12 Union Road, Cambridge CB2 1EZ, UK; fax: (+44)1223-336-033 or deposit@ccdc.cam.uk).

## Figures and Tables

**Table 1:** Asymmetric hydrogenation of acetophenone (**S-1**) employing Ir/**6a** as catalyst.

Entry	M/L	Conversion	*ee*
1	1:3	-	-
2 ^a^	1:2	-	-
3 ^a^	1:3	6%	racemate
4 ^b^	1:3	20%	racemate

^a^ K_2_CO_3_ (20 mol%); ^b^ KO*^t^*Bu (20 mol%).

**Table 2:** Asymmetric hydrogenation of methyl (*Z*)-2-acetamido-3-phenylacrylate (**S-2**) using Ir/**6a** or **6b** as catalyst.

Entry	Solvent	Conversion	*ee*
1 ^a^	CH_2_Cl_2_	98%	8%
2 ^b^	CH_2_Cl_2_	>99%	9%
3 ^b^	MeOH	90%	8%
4 ^b^	THF	50%	-
5 ^c^	CH_2_Cl_2_	95%	8%

Reaction conditions: [Ir(COD)Cl]_2_/**6a** (1:2), substrate/catalyst (S/C) = 100, [substrate] = 0.5 mmol, H_2_ (50 bar), solvent = dichloromethane, 30 °C, 15 h. ^a^ 5 mol% catalyst (M:**6a** = 1:2); ^b^ **6a** (1 mol%); ^c^ **6b** (1 mol%).

## Data Availability

The data presented in this study are available in the Supplementary Materials.

## Supplementary Materials

The following supporting information can be downloaded at: https://www.mdpi.com/article/10.3390/molecules28176210/s1, NMR spectra of **5a**,**b** and **6a**,**b**, details for the crystallographic characterization, HPLC data of **5a** as well as chromatograms from catalytic tests are available in the Supplementary Materials.

## References

[1] Dutartre M., Bayardon J., Jugé S. (2016). Applications and stereoselective syntheses of P-chirogenic phosphorus compounds. Chem. Soc. Rev..

[2] Imamoto T. (2021). Synthesis and applications of high-performance P-chiral phosphine ligands. Proc. Jpn. Acad. Ser. B Phys. Biol. Sci..

[3] Cabré A., Verdaguer X., Riera A. (2022). Recent Advances in the Enantioselective Synthesis of Chiral Amines via Transition Metal-Catalyzed Asymmetric Hydrogenation. Chem. Rev..

[4] Kamer P.C.J., van Leeuwen P.W.N.M. (2012). Phosphorus(III) Ligands in Homogeneous Catalysis.

[5] van Leeuwen P.W.N.M., Kamer P.C.J., Claver C., Pàmies O., Diéguez M. (2011). Phosphite-containing ligands for asymmetric catalysis. Chem. Rev..

[6] Tang W., Zhang X. (2003). New chiral phosphorus ligands for enantioselective hydrogenation. Chem. Rev..

[7] Xu G., Senanayake C.H., Tang W. (2019). P-Chiral Phosphorus Ligands Based on a 2,3-Dihydrobenzo d1,3oxaphosphole Motif for Asymmetric Catalysis. Acc. Chem. Res..

[8] Fu W., Tang W. (2016). Chiral Monophosphorus Ligands for Asymmetric Catalytic Reactions. ACS Catal..

[9] Horner L., Siegel H., Büthe H. (1968). Asymmetric Catalytic Hydrogenation with an Optically Active Phosphinerhodium Complex in Homogeneous Solution. Angew. Chem. Int. Ed..

[10] Knowles W.S., Sabacky M.J. (1968). Catalytic asymmetric hydrogenation employing a soluble, optically active, rhodium complex. Chem. Commun..

[11] Vineyard B.D., Knowles W.S., Sabacky M.J., Bachman G.L., Weinkauff D.J. (1977). Asymmetric hydrogenation. Rhodium chiral bisphosphine catalyst. J. Am. Chem. Soc..

[12] Knowles W.S. (2002). Asymmetric hydrogenations (Nobel lecture). Angew. Chem. Int. Ed..

[13] Noyori R. (2002). Asymmetric Catalysis: Science and Opportunities (Nobel Lecture). Angew. Chem. Int. Ed..

[14] Sandoval C.A., Ohkuma T., Muñiz K., Noyori R. (2003). Mechanism of asymmetric hydrogenation of ketones catalyzed by BINAP/1,2-diamine-rutheniumII complexes. J. Am. Chem. Soc..

[15] Akutagawa S. (1995). Asymmetric synthesis by metal BINAP catalysts. Appl. Catal. A Gen..

[16] Li J.J., Li J.J. (2021). Noyori Asymmetric Hydrogenation. Name Reactions.

[17] Seo C.S.G., Morris R.H. (2019). Catalytic Homogeneous Asymmetric Hydrogenation: Successes and Opportunities. Organometallics.

[18] Biosca M., Salomó E., de La Cruz-Sánchez P., Riera A., Verdaguer X., Pàmies O., Diéguez M. (2019). Extending the Substrate Scope in the Hydrogenation of Unfunctionalized Tetrasubstituted Olefins with Ir-P Stereogenic Aminophosphine-Oxazoline Catalysts. Org. Lett..

[19] Andersson P.G. (2011). Iridium Catalysis.

[20] Hu X.-P., Wang D.-S., Yu C.-B., Zhou Y.-G., Zheng Z., Ma S. (2011). Adventure in Asymmetric Hydrogenation: Synthesis of Chiral Phosphorus Ligands and Asymmetric Hydrogenation of Heteroaromatics. Asymmetric Catalysis from a Chinese Perspective.

[21] Akiyama T., Ojima I. (2022). Catalytic Asymmetric Synthesis.

[22] Mazuela J., Verendel J.J., Coll M., Schäffner B., Börner A., Andersson P.G., Pàmies O., Diéguez M. (2009). Iridium phosphite-oxazoline catalysts for the highly enantioselective hydrogenation of terminal alkenes. J. Am. Chem. Soc..

[23] Crabtree R. (1979). Iridium compounds in catalysis. Acc. Chem. Res..

[24] Roseblade S.J., Pfaltz A. (2007). Iridium-catalyzed asymmetric hydrogenation of olefins. Acc. Chem. Res..

[25] Helmchen G., Pfaltz A. (2000). Phosphinooxazolines—A new class of versatile, modular P,N-ligands for asymmetric catalysis. Acc. Chem. Res..

[26] von Matt P., Pfaltz A. (1993). Chiral Phosphinoaryldihydrooxazoles as Ligands in Asymmetric Catalysis: Pd-Catalyzed Allylic Substitution. Angew. Chem. Int. Ed..

[27] Busacca C.A., Grossbach D., So R.C., O’Brien E.M., Spinelli E.M. (2003). Probing electronic effects in the asymmetric Heck reaction with the BIPI ligands. Org. Lett..

[28] Carroll M.P., Guiry P.J. (2014). P,N ligands in asymmetric catalysis. Chem. Soc. Rev..

[29] Elsevier C.J., de Vries J.G. (2007). The Handbook of Homogeneous Hydrogenation.

[30] Helmchen G., Kudis S., Sennhenn P., Steinhagen H. (1997). Enantioselective catalysis with complexes of asymmetric P,N-chelate ligands. Pure Appl. Chem..

[31] Peters B.B.C., Zheng J., Birke N., Singh T., Andersson P.G. (2022). Iridium-catalyzed enantioconvergent hydrogenation of trisubstituted olefins. Nat. Commun..

[32] Frank D.J., Franzke A., Pfaltz A. (2013). Asymmetric hydrogenation using rhodium complexes generated from mixtures of monodentate neutral and anionic phosphorus ligands. Chem. Eur. J..

[33] Minnaard A.J., Feringa B.L., Lefort L., de Vries J.G. (2007). Asymmetric hydrogenation using monodentate phosphoramidite ligands. Acc. Chem. Res..

[34] Giacomina F., Meetsma A., Panella L., Lefort L., de Vries A.H.M., de Vries J.G. (2007). High enantioselectivity is induced by a single monodentate phosphoramidite ligand in iridium-catalyzed asymmetric hydrogenation. Angew. Chem. Int. Ed..

[35] Komarov I.V., Börner A. (2001). Highly Enantioselective or Not?—Chiral Monodentate Monophosphorus Ligands in the Asymmetric Hydrogenation. Angew. Chem. Int. Ed..

[36] Cabré A., Riera A., Verdaguer X. (2020). P-Stereogenic Amino-Phosphines as Chiral Ligands: From Privileged Intermediates to Asymmetric Catalysis. Acc. Chem. Res..

[37] Xie X., Li S., Chen Q., Guo H., Yang J., Zhang J. (2022). Synthesis and application of novel P-chiral monophosphorus ligands. Org. Chem. Front..

[38] Murai T. (2023). Axis-to-Center Chirality Transfer Reactions of Phosphates with a Binaphthyl Group and their Congeners: New Synthetic Routes to P-Chirogenic Organophosphorus Compounds. Chem. Lett..

[39] Mathey F. (1988). The organic chemistry of phospholes. Chem. Rev..

[40] Wonneberger P., König N., Kraft F.B., Sárosi M.B., Hey-Hawkins E. (2019). Access to 1-Phospha-2-azanorbornenes by Phospha-aza-Diels-Alder Reactions. Angew. Chem. Int. Ed..

[41] Ramazanova K., Lönnecke P., Hey-Hawkins E. (2023). Facile Synthesis of Enantiomerically Pure P-Chiral 1-Alkoxy-2,3-dihydrophospholes via Nucleophilic P-N Bond Cleavage of a 1-Phospha-2-azanorbornene. Chem. Eur. J..

[42] Wonneberger P., König N., Sárosi M.B., Hey-Hawkins E. (2021). Reductive Rearrangement of a 1-Phospha-2-azanorbornene. Chem. Eur. J..

[43] Möller T., Sárosi M.B., Hey-Hawkins E. (2012). Asymmetric phospha-Diels-Alder reaction: A stereoselective approach towards P-chiral phosphanes through diastereotopic face differentiation. Chem. Eur. J..

[44] Möller T., Wonneberger P., Kretzschmar N., Hey-Hawkins E. (2014). P-chiral phosphorus heterocycles: A straightforward synthesis. Chem. Commun..

[45] Möller T., Wonneberger P., Sárosi M.B., Coburger P., Hey-Hawkins E. (2016). P-chiral 1-phosphanorbornenes: From asymmetric phospha-Diels-Alder reactions towards ligand design and functionalisation. Dalton Trans..

[46] Denmark S.E., Hammer R.P., Weber E.J., Habermas K.L. (1987). Diphenylmethylsilyl ether (DPMS): A protecting group for alcohols. J. Org. Chem..

[47] Bols M., Pedersen C.M. (2017). Silyl-protective groups influencing the reactivity and selectivity in glycosylations. Beilstein J. Org. Chem..

[48] Crouch R.D. (2013). Recent Advances in Silyl Protection of Alcohols. Synth. Commun..

[49] Wuts P.G., Green T.W. (2007). Greene’s Protective Groups in Organic Synthesis.

[50] Davies J.S., Higginbotham C.L., Tremeer E.J., Brown C., Treadgold R.C. (1992). Protection of hydroxy groups by silylation: Use in peptide synthesis and as lipophilicity modifiers for peptides. J. Chem. Soc. Perkin Trans. 1.

[51] Patschinski P., Zhang C., Zipse H. (2014). The Lewis base-catalyzed silylation of alcohols—A mechanistic analysis. J. Org. Chem..

[52] Corey E.J., Venkateswarlu A. (1972). Protection of hydroxyl groups as tert-butyldimethylsilyl derivatives. J. Am. Chem. Soc..

[53] Weinhold F., West R. (2011). The Nature of the Silicon–Oxygen Bond. Organometallics.

[54] Kaftory M., Kapon M., Botoshansky M., Patai S., Rappoport Z. (1998). The Structural Chemistry of Organosilicon Compounds. The Chemistry of Organic Silicon Compounds.

[55] Wender P.A., Bi F.C., Brodney M.A., Gosselin F. (2001). Asymmetric synthesis of the tricyclic core of NGF-inducing cyathane diterpenes via a transition-metal-catalyzed 5 + 2 cycloaddition. Org. Lett..

[56] de Vries E.F.J., Brussee J., van der Gen A. (1994). Intramolecular Reductive Cleavage of tert-Butyldimethylsilyl Ethers. Selective Mono-Deprotection of Bis-Silyl-Protected Diols. J. Org. Chem..

[57] Crouch R.D. (2013). Selective deprotection of silyl ethers. Tetrahedron.

[58] Pfaltz A., Blankenstein J., Hilgraf R., Hörmann E., McIntyre S., Menges F., Schönleber M., Smidt S.P., Wüstenberg B., Zimmermann N. (2003). Iridium-Catalyzed Enantioselective Hydrogenation of Olefins. Adv. Synth. Catal..

[59] Peters B.B.C., Andersson P.G. (2022). The Implications of the Brønsted Acidic Properties of Crabtree-Type Catalysts in the Asymmetric Hydrogenation of Olefins. J. Am. Chem. Soc..

[60] Harris R.K., Becker E.D., Cabral de Menezes S.M., Goodfellow R., Granger P. (2002). NMR Nomenclature: Nuclear Spin Properties and Conventions for Chemical Shifts. IUPAC Recommendations 2001. Solid State Nucl. Magn. Reson..

[61] Rigaku Corporation (1995–2023). CrysAlisPro Software System.

[62] Sheldrick G.M. (2015). SHELXT—Integrated space-group and crystal-structure determination. Acta. Cryst. Sect. A Found. Adv..

[63] Sheldrick G.M. (2015). Crystal structure refinement with SHELXL. Acta Cryst. Sect. C Struct. Chem..

[64] Brandenburg K. Crystal Impact GbR.

