# Supplementary material for: Access to Enantiomerically Pure P-Chiral 1-Phosphanorbornane Silyl Ethers
Source: Molecules. 2023 Aug 23;28(17):6210. doi: 10.3390/molecules28176210 (PMC10488433; doi:10.3390/molecules28176210)
Supplement: Supplementary file 1 [file molecules-28-06210-s001.zip › molecules-2574134-supplementary.pdf]

Access to Enantiomerically Pure *P*-chiral 1-Phosphanorbornane Silyl Ethers

Kyzgaldak Ramazanova [1], Soumyadeep Chakrabortty [2], Fabian Kallmeier [2], Nadja Kretzschmar [1], Sergey Tin [2], Peter Lönnecke [1], Johannes G. de Vries[2] and Evamarie Hey-Hawkins [1,*]

[1]Institute of Inorganic Chemistry, Faculty of Chemistry and Mineralogy, Leipzig University, Johannisallee 29, 04103, Leipzig, Germany

[2]Leibniz Institute for Catalysis (LIKAT), Albert-Einstein-Straße 29A, 18059, Rostock, Germany

*Correspondence: hey@uni-leipzig.de

Table of Contents

1. NMR spectra of **5a,b** and **6a,b** 2

2. HPLC data of **5a** 8

3. X-ray crystallography 9

4. NMR spectra of **P2** 11

5. HPLC chromatograms of **P2** 12

1. NMR spectra of **5a,b** and **6a,b**

Numbering scheme:

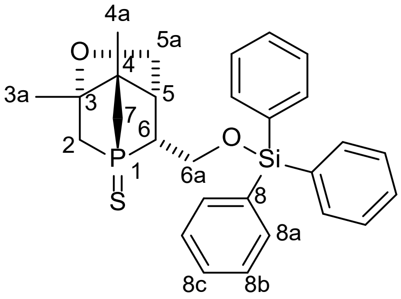

5a

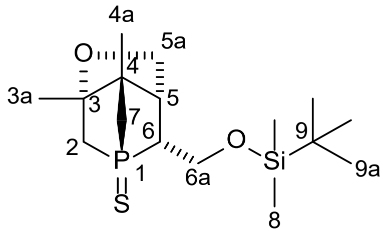

5b

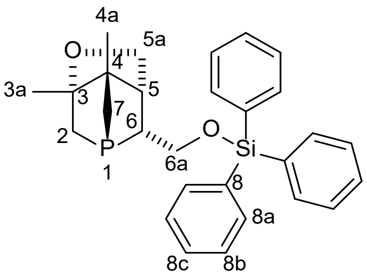

6a

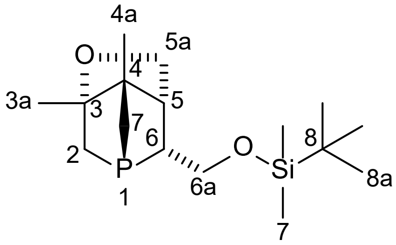

6b

NMR spectra of 5a:

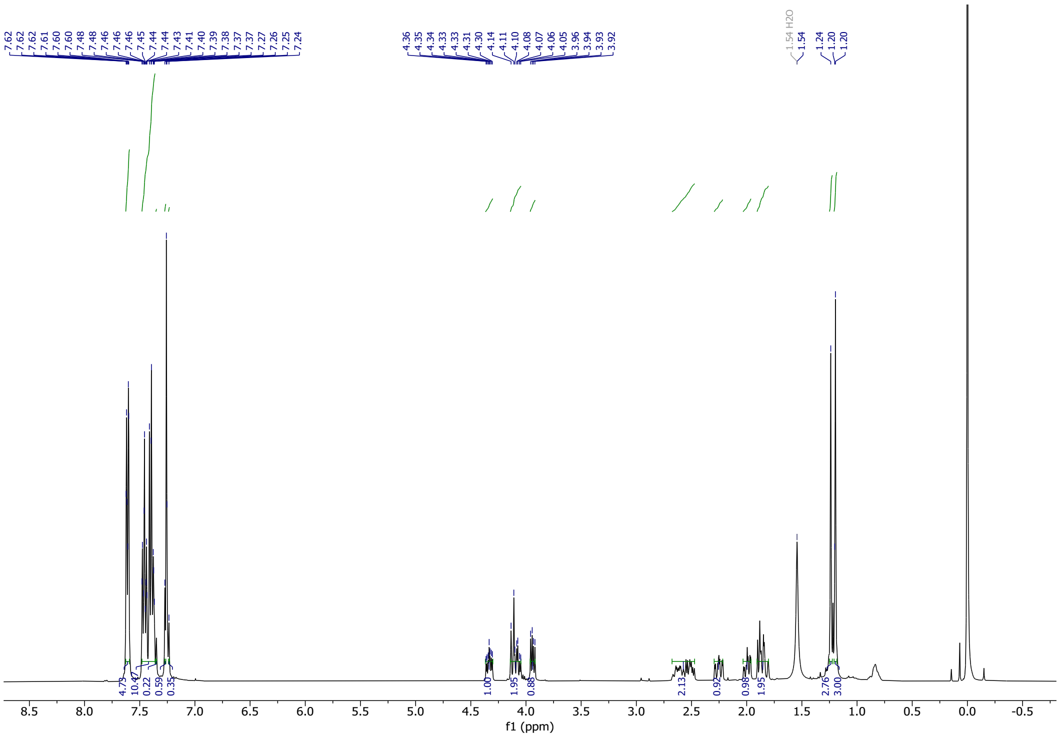

Figure S1. ^{1}H NMR spectrum of **5a** in $CDCl_3$.

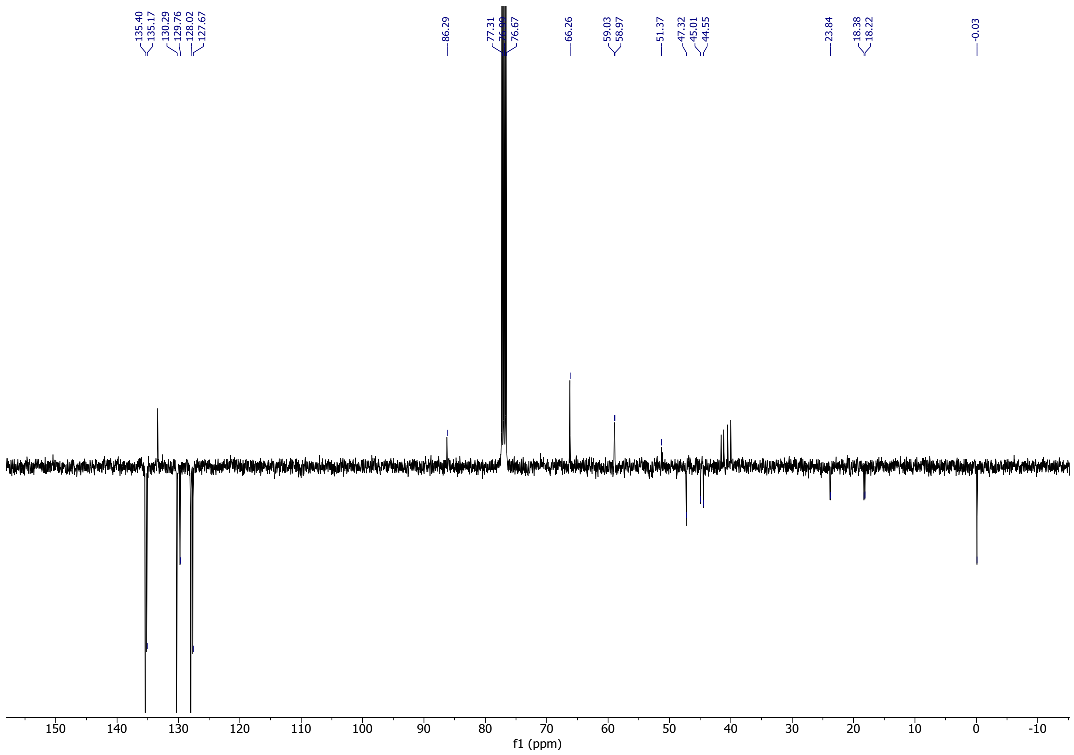

Figure S2. $^{13}C\{^{1}H\}$ APT NMR spectrum of **5a** in $CDCl_3$.

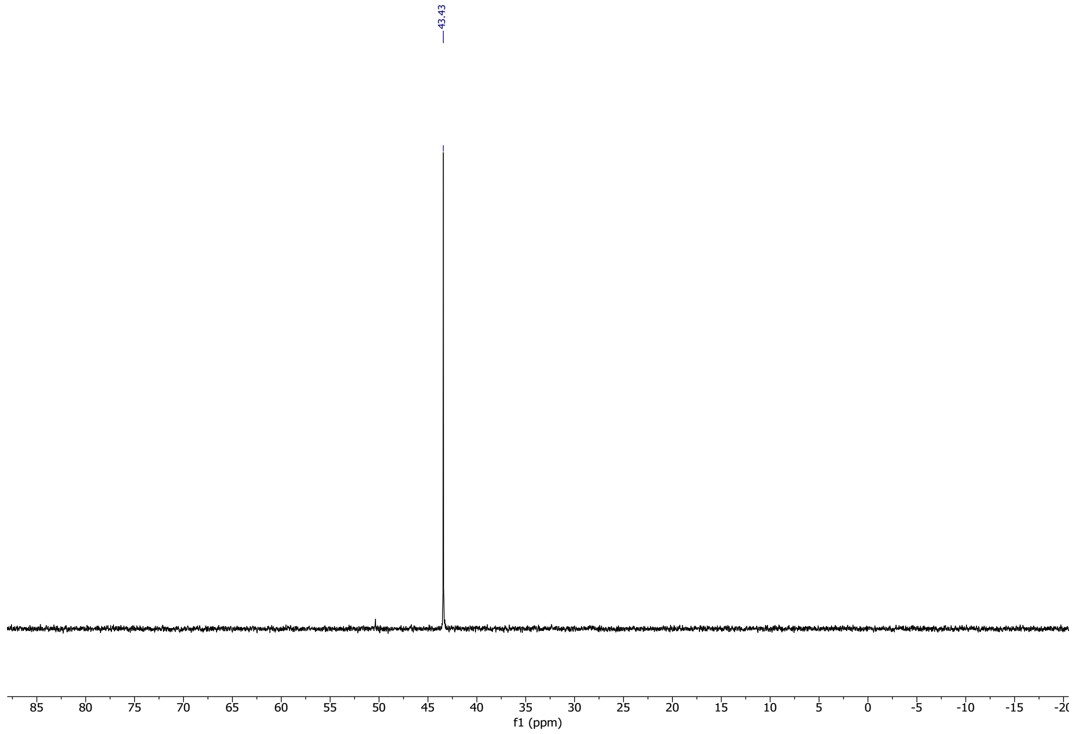

Figure S3. $^{31}P\{^{1}H\}$ NMR spectrum of **5a** in $CDCl_3$.

NMR spectra of 5b:

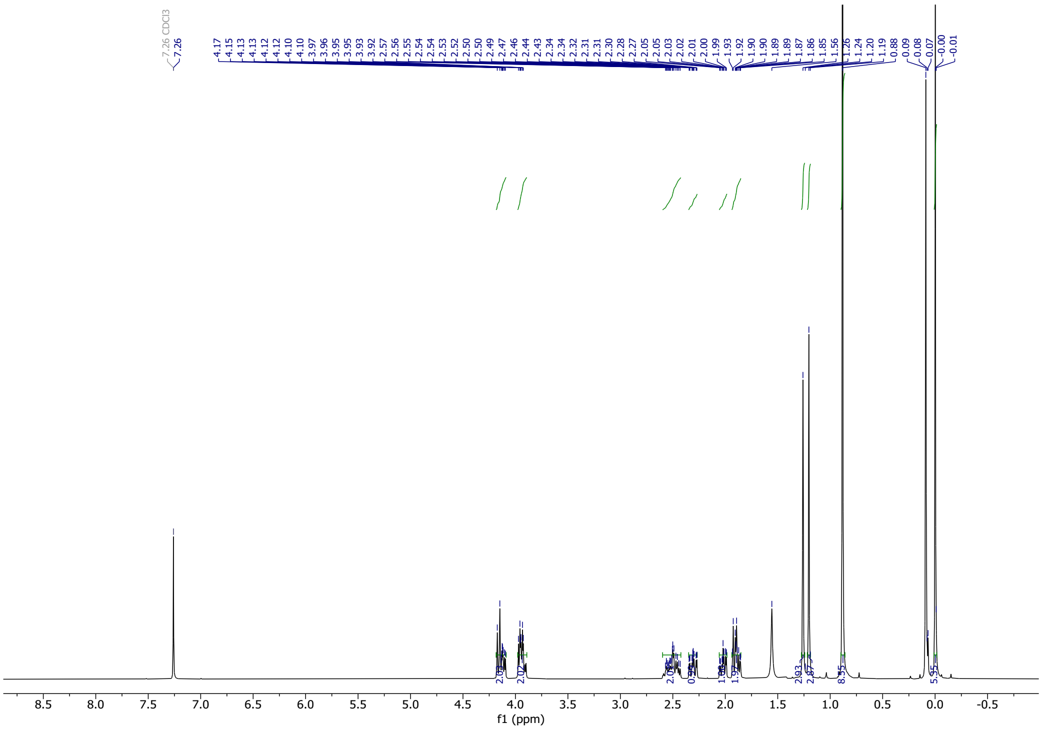

Figure S4. ^{1}H NMR spectrum of **5b** in $CDCl_3$.

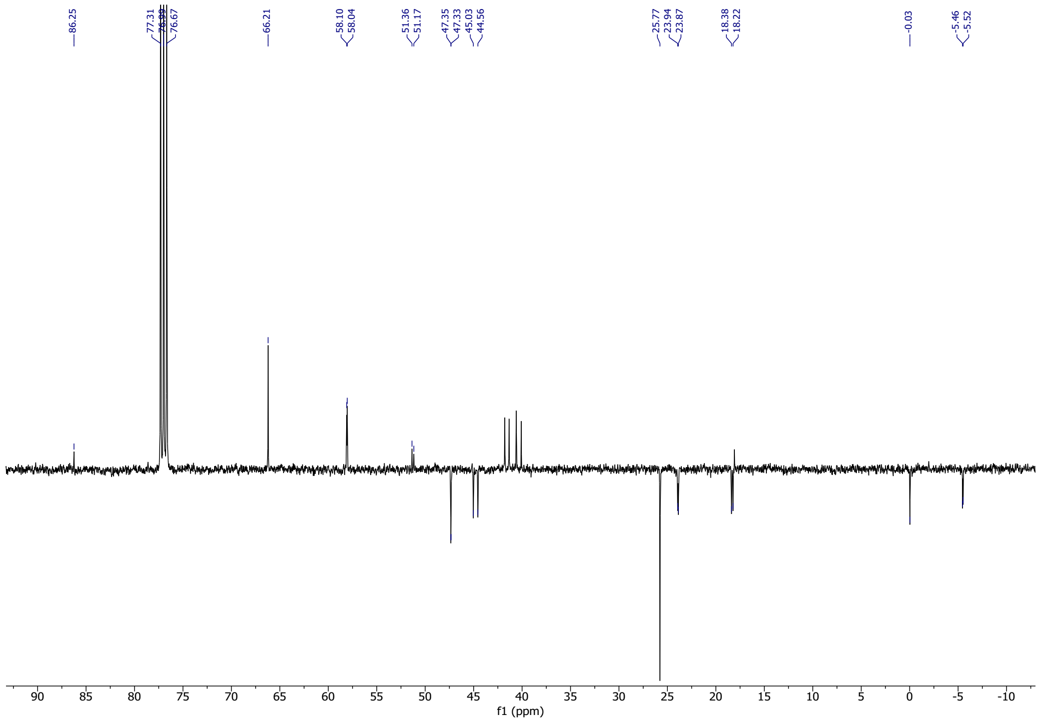

Figure S5. $^{13}C\{^{1}H\}$ APT NMR spectrum of **5b** in $CDCl_3$.

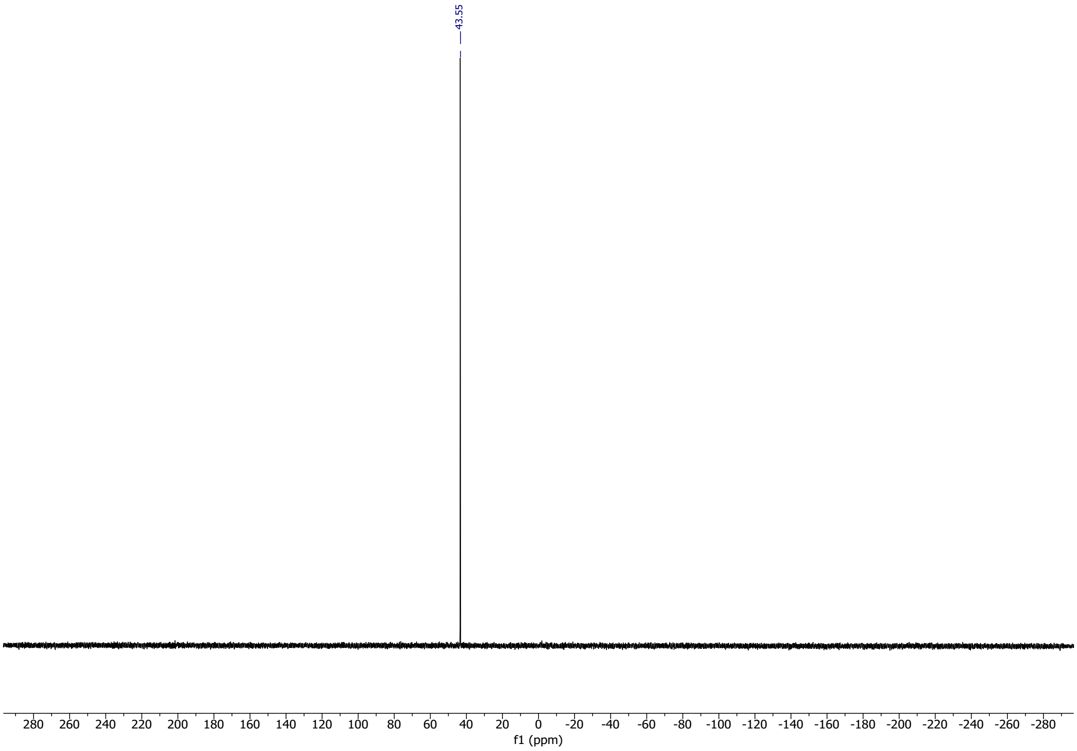

Figure S6. $^{31}P\{^{1}H\}$ NMR spectrum of 5**b** in $CDCl_3$.

NMR spectra of 6a:

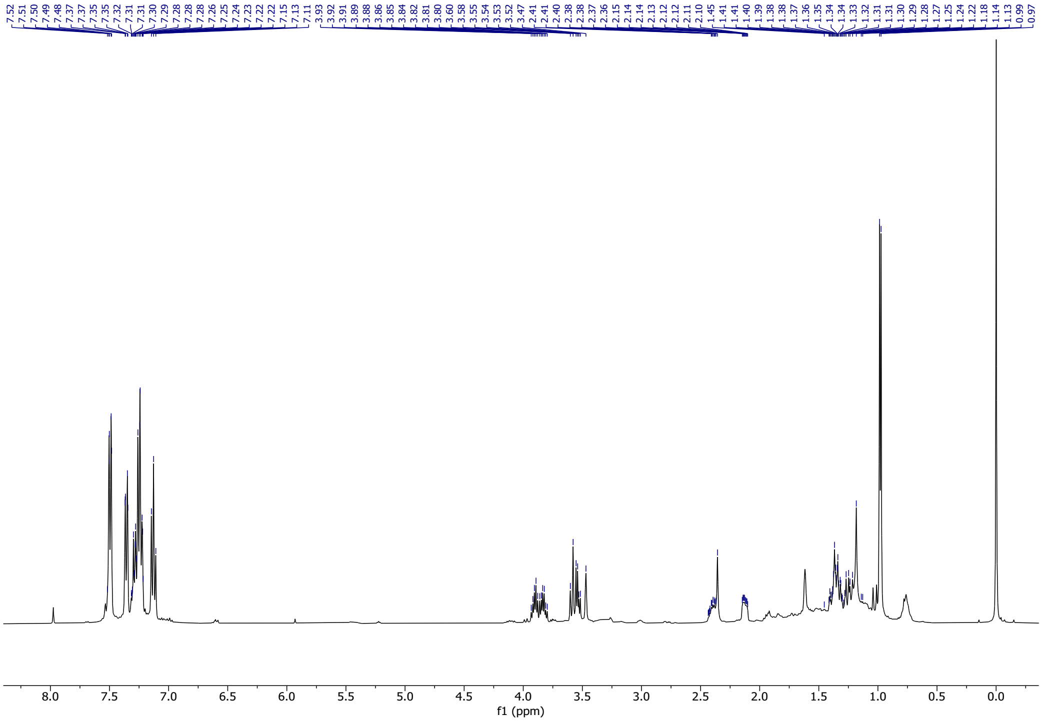

Figure S7. ^{1}H NMR spectrum of **6a** in THF-d_8.

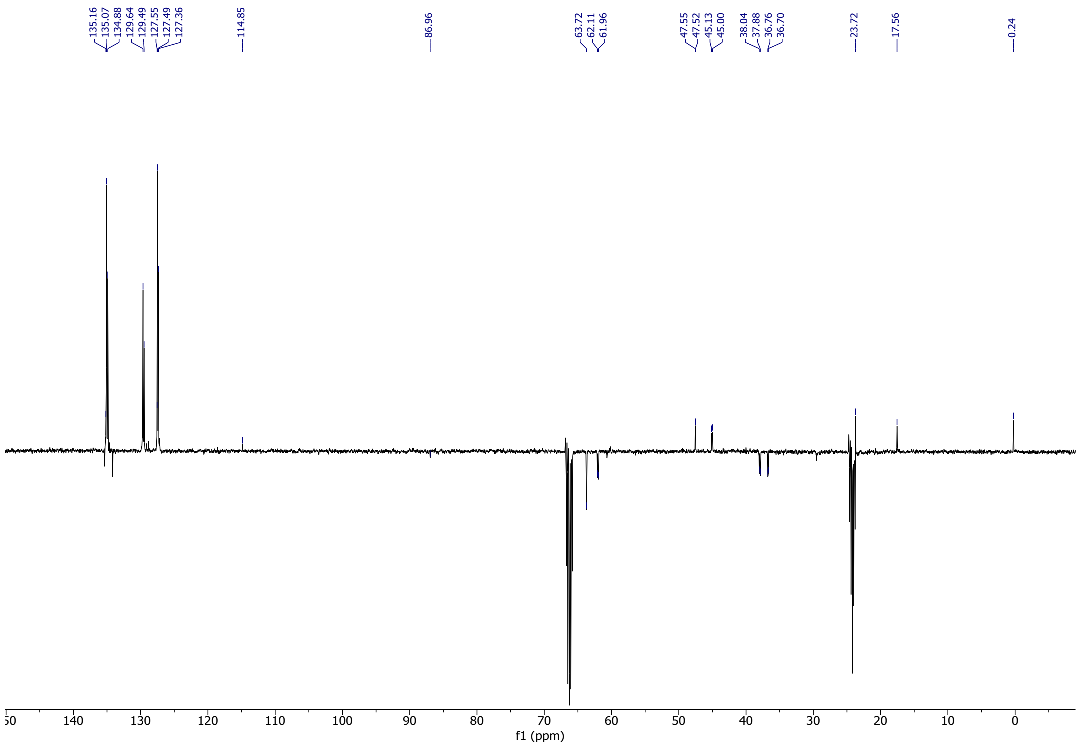

Figure S8. $^{13}C\{^{1}H\}$ APT NMR spectrum of **6a** in THF-d_8.

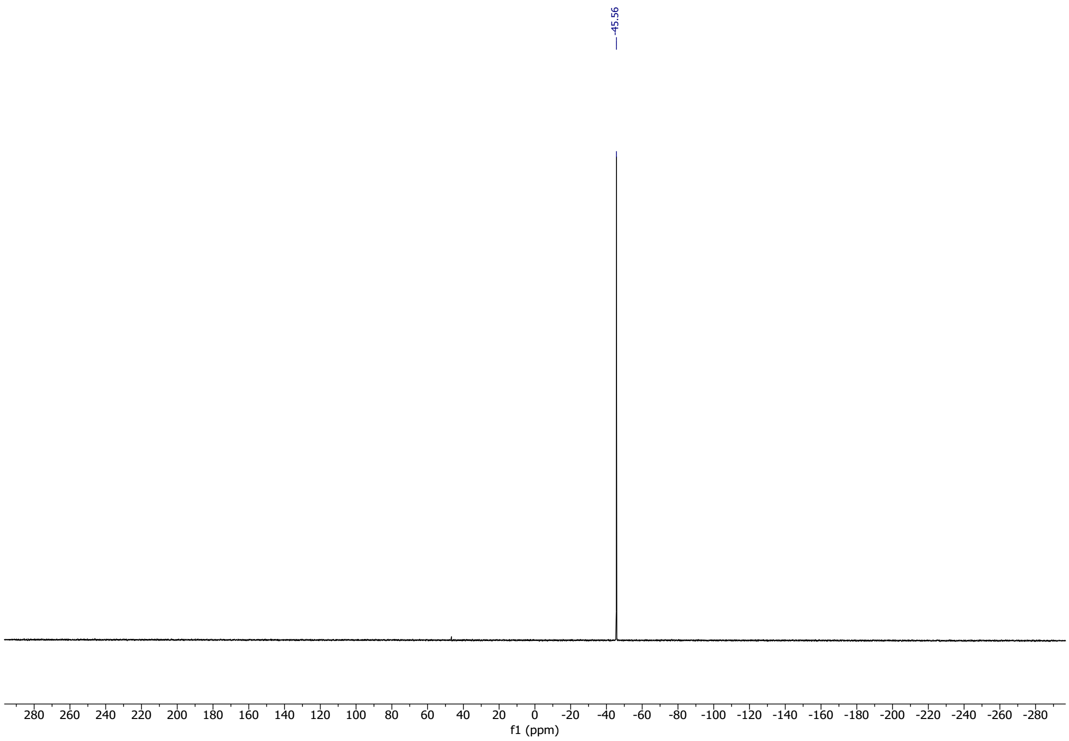

Figure S9. $^{31}P\{^{1}H\}$ NMR spectrum of **6a** in C_6D_6.

NMR spectra of 6b:

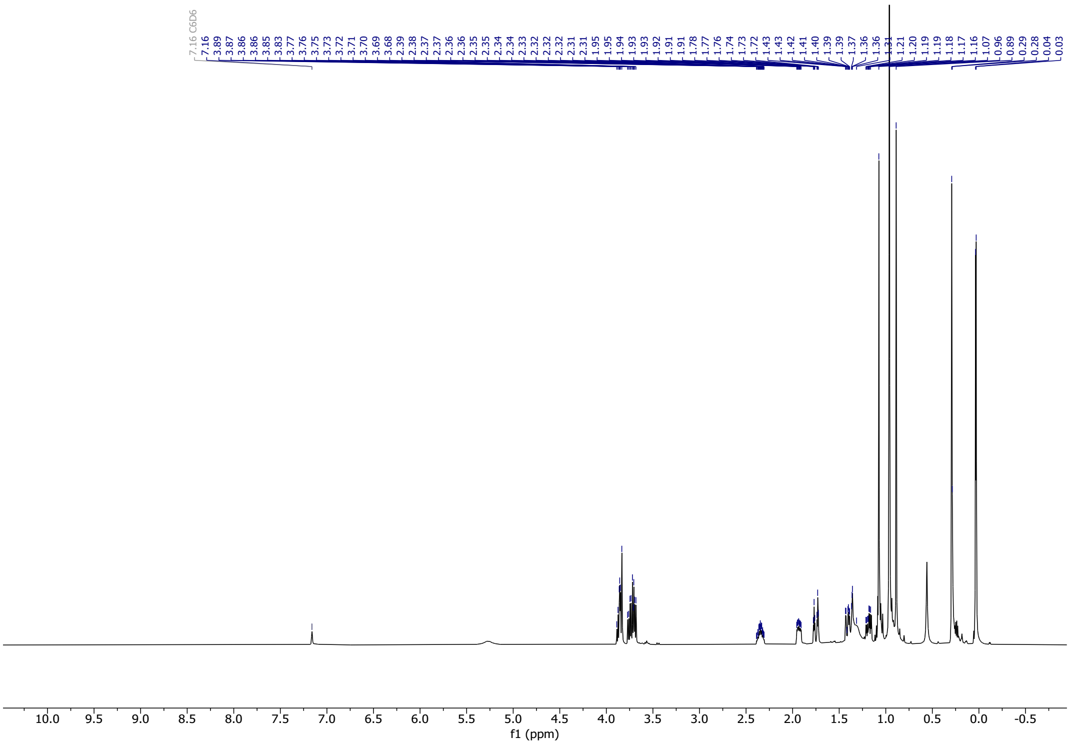

Figure S10. ^{1}H NMR spectrum of **6b** in C_6D_6.

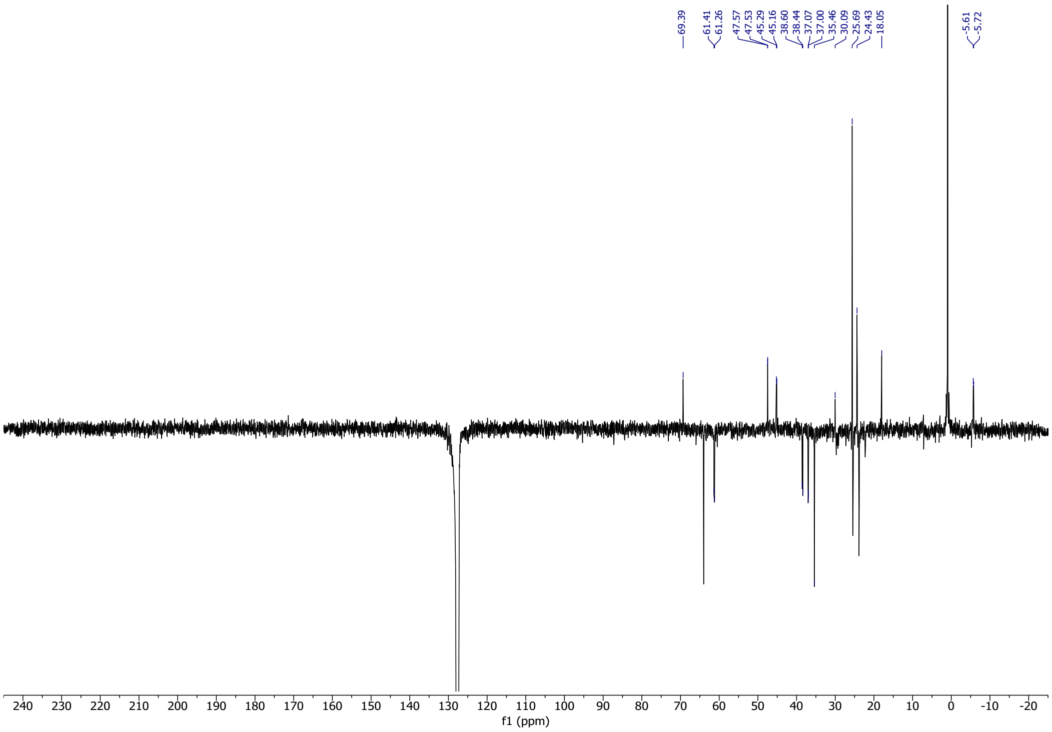

Figure S11. $^{13}C\{^{1}H\}$ APT NMR spectrum of **6b** in C_6D_6.

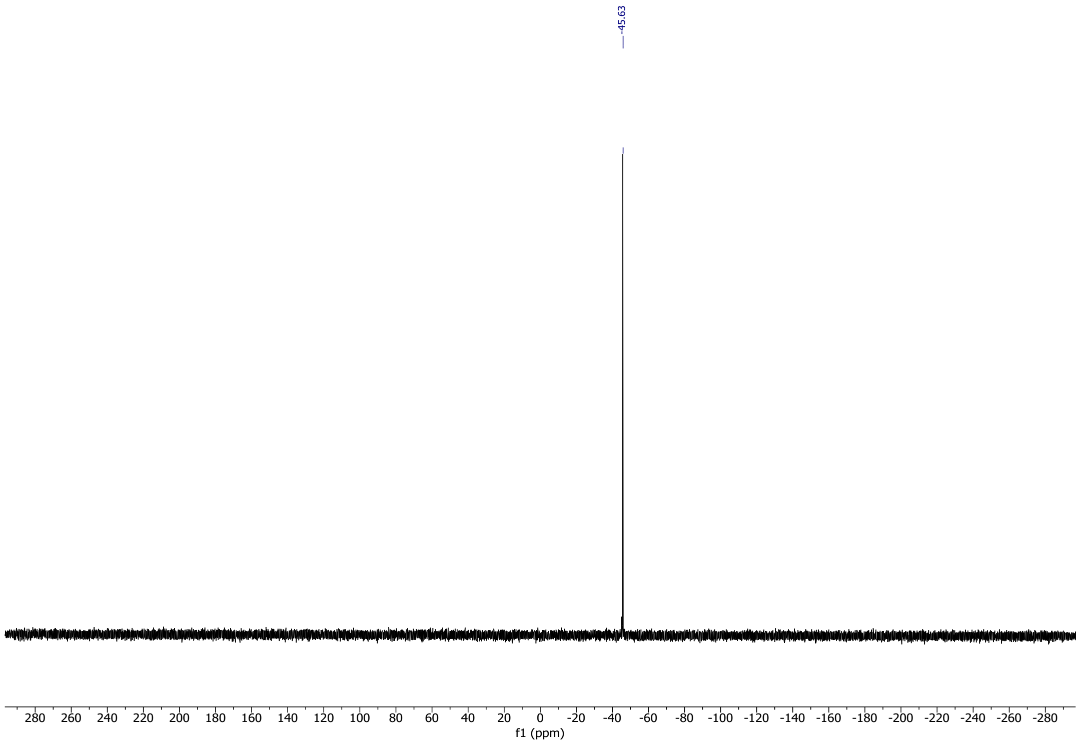

Figure S12. $^{31}P\{^{1}H\}$ NMR spectrum of **6b** in C_6D_6.

2. HPLC data of **5a**

Eluent: 20% ^{i}PrOH, 80% *n*-hexane

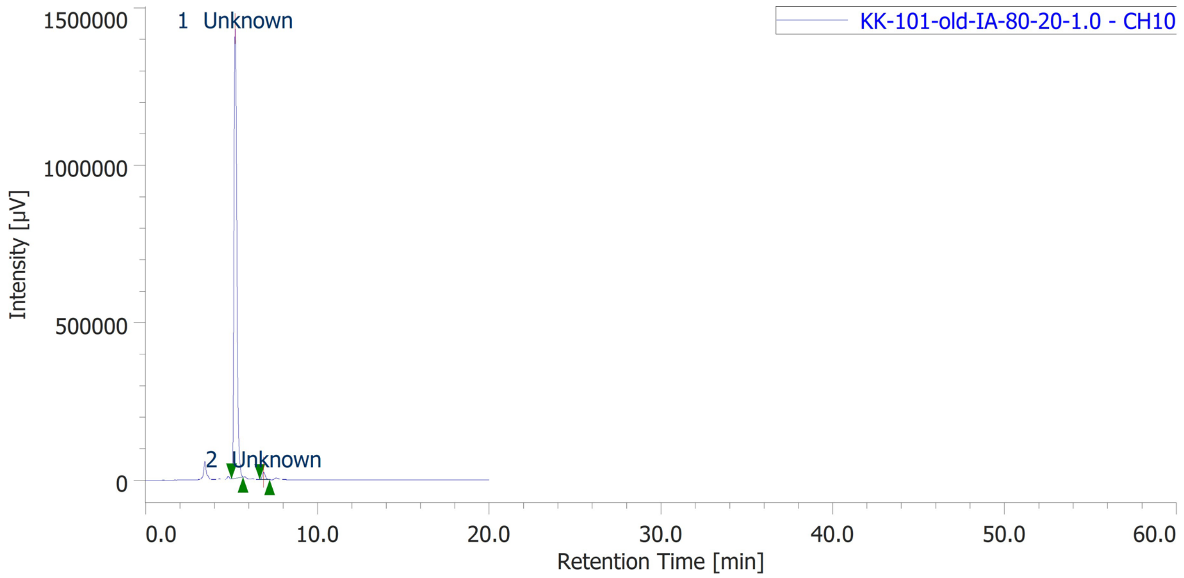

Figure S13. Chromatogram of **5a**.

#	Peak Name	CH	tR [min]	Area [µV·sec]	Height [µV]	Area%	Height%	Quantity	NTP	Resolution	Symmetry Factor	Warning
1	Unknown	10	5.203	16650701	1426840	98.260	98.395	N/A	4594	5.193	1.402	
2	Unknown	10	6.863	294923	23278	1.740	1.605	N/A	6759	N/A	1.158	

3. X-ray crystallography

Table S1. X-ray crystallography data of compounds **5a** and **5b**

Compound	**5a**	**5b**
Empirical formula	$C_{28}H_{31}O_2PSSi$	$C_{16}H_{31}O_2PSSi$
Formula weight	490.65	346.53
Temperature [K]	130(2) K	130(2) K
Wavelength [pm]	71.073 pm	71.073 pm
Crystal system	Triclinic	Monoclinic
Space group	$P1$	$P2_1$
Unit cell dimensions		
a [pm]	a = 897.11(3)	a = 1085.78(4)
b [pm]	b = 1259.36(4)	b = 761.49(2)
c [pm]	c = 1337.86(4)	c = 1172.77(3)
α [deg]	63.086(3)	90
β [deg]	74.155(3)	96.596(3)
γ [deg]	69.466(3)	90
Volume [nm^3]	1.25028(8)	0.96324(5)
Z	2	2
$\rho_{(calculated)}$ [Mg/m^3]	1.303	1.195
μ [mm^{-1}]	0.265	0.316
F(000)	520	376
Crystal size [mm^3]	0.20 · 0.20 · 0.03	0.50 · 0.25 · 0.02
Θ_{Min} / Θ_{Max} [deg]	1.881 / 32.599	1.748 / 32.457
Index ranges	-13 ≤ h ≤ 13<br>-18 ≤ k ≤ 18<br>-20 ≤ l ≤ 20	-15 ≤ h ≤ 16<br>-11 ≤ k ≤ 10<br>-17 ≤ l ≤ 17
Reflections collected	26920	15131
Indp. reflections (R_{int})	15734 (0.0349)	6338 (0.0415)
Completeness (Θ_{Max})	100.0 % (30.510)	100.0 % (30.510)
T_{Max} / T_{Min}	1.00000 / 0.75234	1.00000 / 0.74332

Restraints / parameters	15 / 843	1 / 197
Gof on F^2	1.017	1.022
R1 / wR2 (I>2σ(I))	0.0476 / 0.0930	0.0428 / 0.0859
R1 / wR2 (all data)	0.0659 / 0.1015	0.0570 / 0.0928
Absolute structure parameter	-0.01(4)	0.07(5)
Residual electron density [e·Å^{-3}]	0.324 / -0.360	0.243 / -0.336
CCDC Number	2287331	2287332

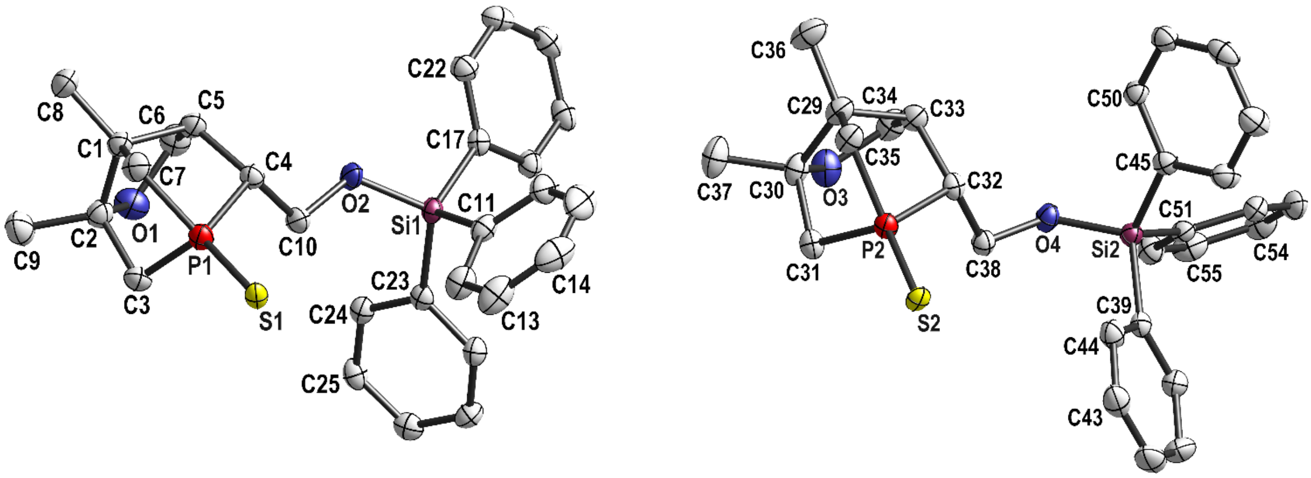

Figure S14. Molecular structure of **5a**. Hydrogen atoms were omitted for clarity. Both independent molecules of **5a** are shown. Displacement ellipsoids are drawn at the 50 % probability level.

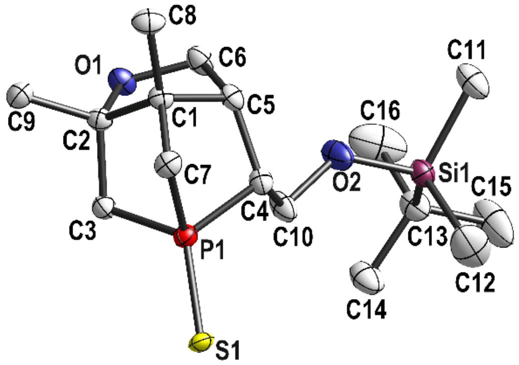

Figure S15. Molecular structure of **5b**. Hydrogen atoms were omitted for clarity. Displacement ellipsoids are drawn at the 50 % probability level.

4. NMR spectra of **P2**

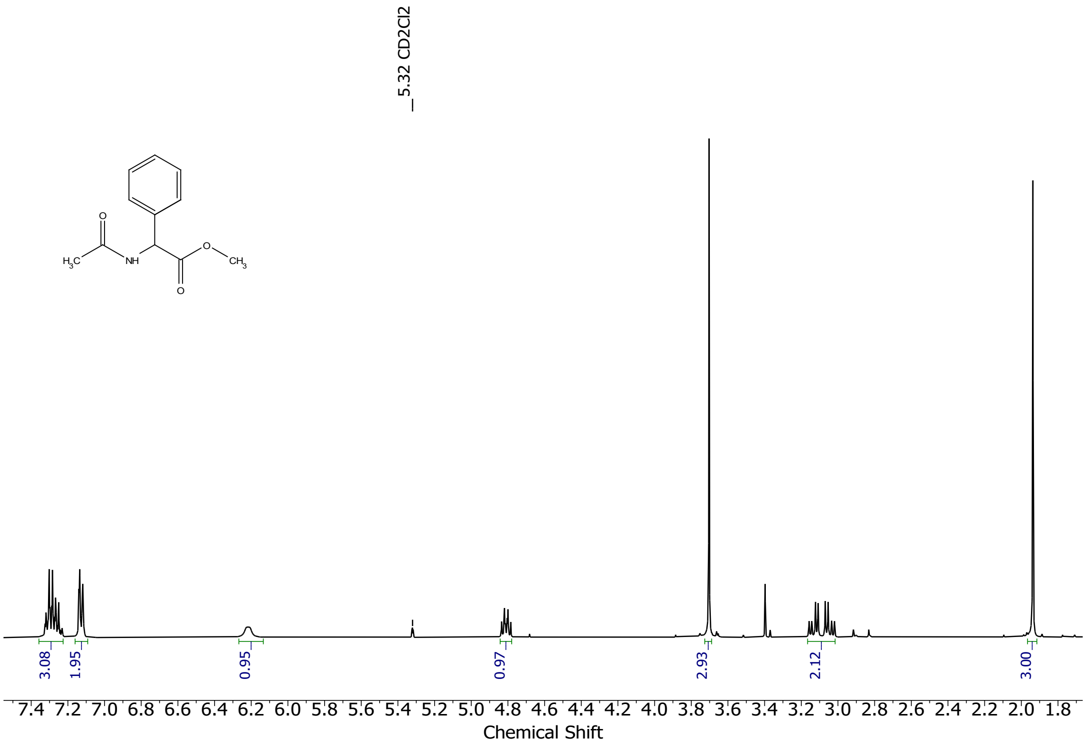

Figure S16. ^{1}H NMR spectrum of **P2** in CD_2Cl_2.

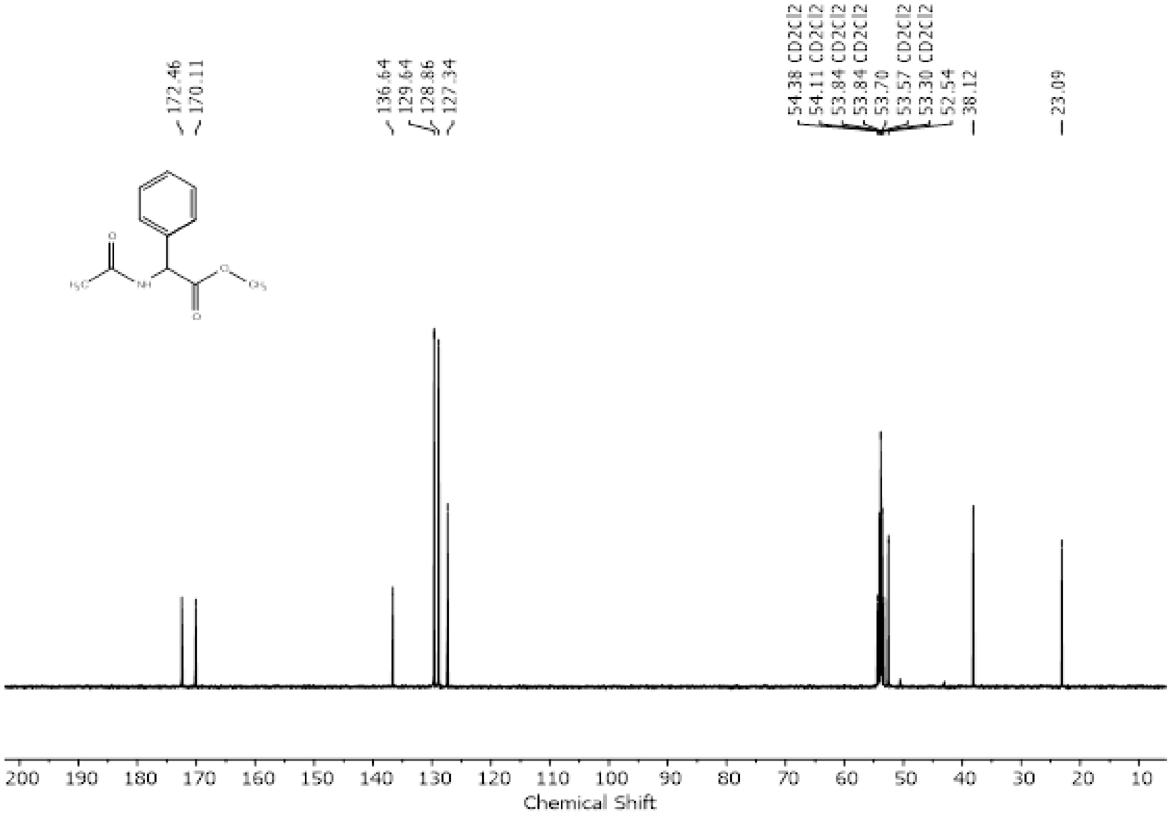

Figure S17. $^{13}C\{^1H\}$ NMR spectrum of **P2** in CD_2Cl_2.

5. GC traces of **P2**

<u>Chiral GC column: 25m Lipodex G</u>

Additional Info : Peak(s) manually integrated

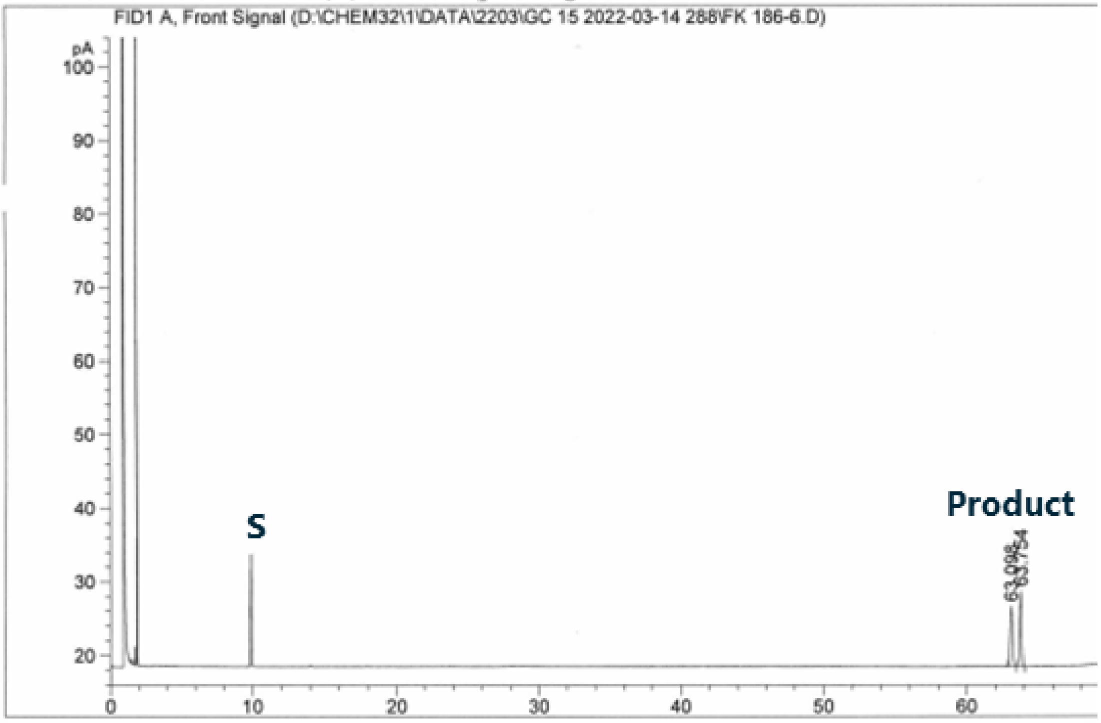

Area Percent Report

Sorted By : Signal
Multiplier : 1.0000
Dilution : 1.0000
Use Multiplier & Dilution Factor with ISTDs

Signal 1: FID1 A, Front Signal

Peak #	RetTime [min]	Type	Width [min]	Area [pA*s]	Height [pA]	Area %
1	63.098	BB	0.1460	97.35970	8.11376	50.01973
2	63.754	BB	0.1183	97.28291	10.10215	49.98027
Totals :				194.64260	18.21591	

Figure S18. Chromatogram of racemic **P2** (S = solvent).

Additional Info : Peak(s) manually integrated

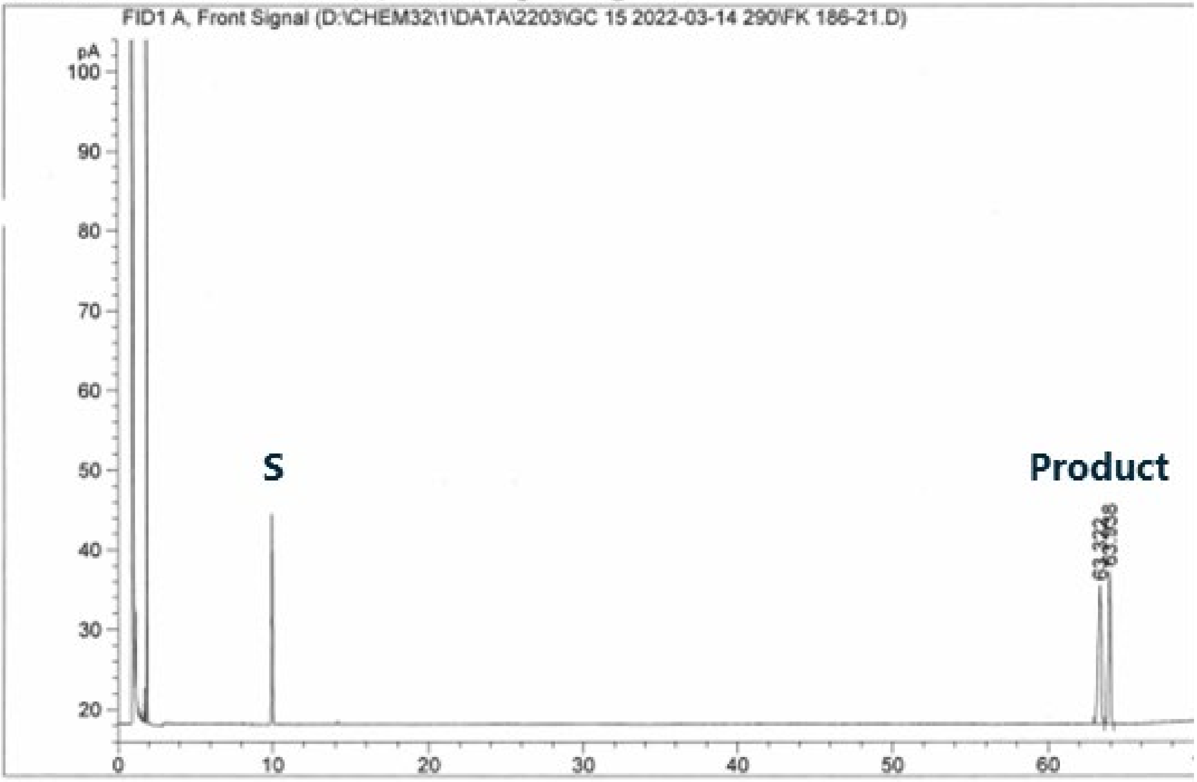

===

Area Percent Report

===

Sorted By : Signal
Multiplier : 1.0000
Dilution : 1.0000
Use Multiplier & Dilution Factor with ISTDs

Signal 1: FID1 A, Front Signal

Peak #	RetTime [min]	Type	Width [min]	Area [pA*s]	Height [pA]	Area %
1	63.322	BV	0.1750	228.11862	17.28439	54.28441
2	63.938	VB	0.1218	192.11002	19.02892	45.71559
Totals :				420.22864	36.31330	

/ 54,30%
/ 45,70%

Figure S19. Chromatogram of enantio-enriched **P2** (Table 2, Entry 2, (S = solvent)).

Additional Info : Peak(s) manually integrated

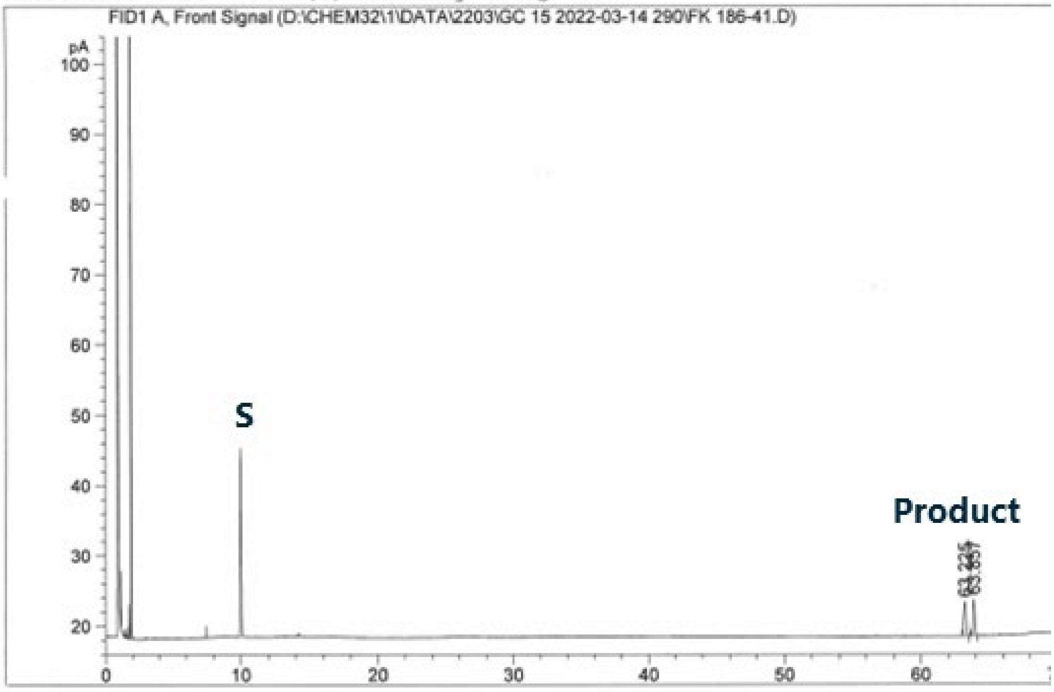

===
Area Percent Report
===

Sorted By : Signal
Multiplier : 1.0000
Dilution : 1.0000
Use Multiplier & Dilution Factor with ISTDs

Signal 1: FID1 A, Front Signal

Peak #	RetTime [min]	Type	Width [min]	Area [pA*s]	Height [pA]	Area %
1	63.225	BB	0.1397	54.31665	4.77688	53.61098
2	63.857	BB	0.1192	46.99963	5.11480	46.38902
Totals :				101.31628	9.89168	

/53,53%
/46,47%

Figure S20. Chromatogram of enantio-enriched **P2** (Table 2, Entry 5, (S = solvent)).
